# Contributions of action potentials to scalp EEG: Theory and biophysical simulations

**DOI:** 10.1371/journal.pcbi.1012794

**Published:** 2025-02-04

**Authors:** Niklas Brake, Anmar Khadra

**Affiliations:** 1 Quantitative Life Sciences PhD Program, McGill University, Montreal, Quebec, Canada; 2 Department of Physiology, McGill University, Montreal, Quebec, Canada; University of California, San Diego, UNITED STATES OFAMERICA

## Abstract

Differences in the apparent 1/f component of neural power spectra require correction depending on the underlying neural mechanisms, which remain incompletely understood. Past studies suggest that neuronal spiking produces broadband signals and shapes the spectral trend of invasive macroscopic recordings, but it is unclear to what extent action potentials (APs) influence scalp EEG. Here, we combined biophysical simulations with statistical modelling to examine the amplitude and spectral content of scalp potentials generated by the electric fields from spiking activity. In physiological parameter regimes, we found that APs contribute negligibly to the EEG spectral trend. Consistent with this, comparing our biophysical simulations with previously published data from pharmacologically paralyzed subjects suggested that the EEG spectral trend can be explained by a combination of synaptic timescales and electromyogram contamination. We also modelled rhythmic EEG generation, finding that APs can generate detectable narrowband power between approximately 60 and 1000 Hz, reaching frequencies much faster than would be possible from synaptic currents. Finally, we show that different spectral detrending strategies are required for AP generated oscillations compared to synaptically generated oscillations, suggesting that existing detrending methods for EEG spectra need to be modified for high frequency signals.

## Author summary

Electroencephalography (EEG) allows researchers and clinicians to measure millisecond changes in human brain activity without surgery or other invasive procedures. However, the neural mechanisms that produce EEG signals remain only partially understood. While brain rhythms have been extensively characterized with EEG, scalp recordings also exhibit non-rhythmic, broadband signals, which are only beginning to be understood. It is known that in recordings made inside brain tissue, neuronal spiking activity produces broadband signals at high frequencies. Here, we performed detailed biophysical and computational modelling of electric field generation in the brain to ascertain the extent to which spiking activity contributes to scalp EEG. We find that it generally does not, suggesting that high frequency broadband EEG signals reflect noise unrelated to brain activity, and validating that low frequency broadband signals are produced by electrical transmission between neurons and not spiking activity. However, our results do characterize a range of frequencies where EEG oscillations may be generated, either in part or in full, by spiking activity. We conclude that spiking activity does not produce broadband signals, but can still generate narrowband signals at high frequencies. Understanding the origins of high-frequency EEG signals has important implications for interpreting scalp recordings and informs the design of quantitative methods for signal analysis.

## Introduction

Understanding the neural mechanisms underlying EEG generation is important for inferring changes in brain state, as well as developing methods to filter out irrelevant signals. Towards this latter aim, recent work has focused on characterizing the neural basis of broadband EEG signals and defining when and how EEG spectra need to be detrended [1–3]. Studies into the neural basis of broadband EEG have primarily focused on synaptic filtering [3–6] and low frequency, aperiodic network fluctuations [3,7,8]. However, in addition to synaptic contributions, the spectral trend observed in invasive, large-scale neural recordings, such as the local field potential (LFP) [9–11] and intracranial EEG (iEEG) [12,13] including electrocorticography (ECoG) [14–17], is believed to reflect broadband contributions from spiking activity [9,10,18], especially at frequencies above  ~ 60 *Hz*, the so-called high gamma range. Such high frequency broadband contributions are thought to be important for determining the slope of the 1 ∕ *f* spectral trend [19].

In comparison to invasive recording techniques, the majority of the unprocessed EEG signal above 30 *Hz* reflects muscle activity [20–23]. Moreover, EEG is thought to be incapable of measuring APs because they are believed to be too brief and unsynchronized [24,25]. Nonetheless, when muscle artifacts are corrected for, EEG recordings have displayed transients in the high gamma range [26–28], similar to those observed in LFP and iEEG recordings. If such high frequency transients are indeed generated by synchronized APs, it would hold significant implication for interpreting spectral peaks and correcting for the EEG spectral trend. Interestingly, a recent biophysical modelling study showed that APs account for almost 20% of the amplitude of single-neuron dipoles, and concluded that APs can contribute significantly to EEG rhythms [29]. However, a systemic investigation into the ability of APs to produce detectable scalp potentials has not been undertaken. Additionally, the potential contribution of APs to aperiodic EEG signals and the overall spectral trend has not been explored.

In this study, we aim to address this gap by employing a quantitative approach that explores AP-generated EEG signals, a type of signal that we refer to hereafter as apEEG for brevity. To begin, we employ a combination of biophysical simulations and statistical modelling to examine the impact of single neuron properties and spike synchrony on the amplitude and spectral features of apEEG signals. Using these results, we evaluate whether apEEG can exhibit experimentally-measurable narrowband and broadband high gamma power. Our results have implications for interpreting high frequency EEG rhythms and for designing practical methods for spectral detrending.

## Results

### Unitary AP response of single-neuron dipoles is approximately linear

**Fig 1:**
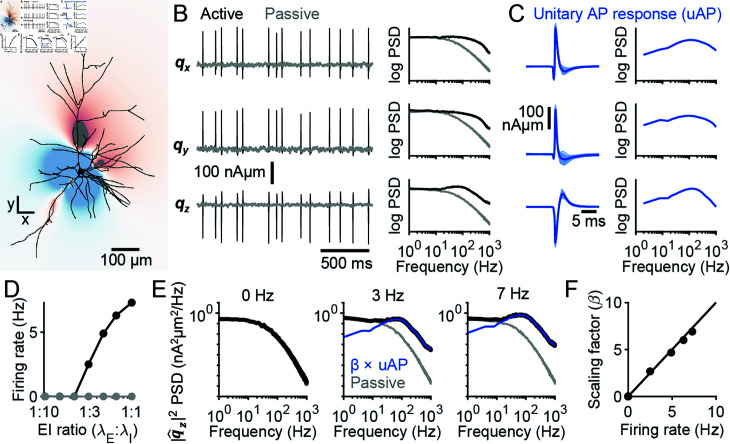
Calculating the unitary AP response. (A) The extracellular electric field generated by a neuron is shown at the peak of an AP. (B) Left: The single-neuron dipole, , associated with the neuron in panel A for the active model (black) and passive model with sodium channels removed (grey). The x, y, and z components of the vector are plotted from top to bottom. Notice the correspondence in the subthreshold fluctuations between the two sets of simulations. Right: The power spectrum of each dipole component trace on the left for the active (black) and passive (gray) model. (C) Left: The difference between the single-neuron dipoles calculated with the active and passive model aligned to each AP (light blue), along with the spike-triggered average (dark blue) which defines the unitary AP response. x, y, and z components are shown from top to bottom. Right: the power spectrum of the unitary AP response. (D) The firing rate of the active (black) and passive (gray) model as a function of E:I ratio, defined as the ratio between the rate of excitatory synapse activation, λ_*E*_, to that of inhibitory synapse activation, λ*I*. (E) The power spectrum of the z component of the single-neuron dipole (black) at three different firing frequencies: 0 *Hz* (left), 3 *Hz* (middle) and 7 *Hz* (right), along with the spectra of the passive models (gray) and the spectra of the unitary AP response (blue) shown previously in panel C. Notice how the unitary AP spectrum matches, up to a scaling factor (*β*), the single-neuron dipole spectrum at high frequency. (F) The scaling factor for the unitary AP spectrum that fits the single-neuron dipole spectrum, plotted as a function of the firing rate (black dots). These data points almost align perfectly with the unity line (black line). The x and y components of the dipole vector show the same behaviour (S1 Fig).

The contributions of an individual neuron to the electric potential measured by a distant electrode can be modelled by a single dipole vector that varies with time [30,31]. This case applies well to EEG signals due to the distance between the brain and scalp electrodes. To understand how APs contribute to EEG, we therefore first sought to characterize the contributions of APs to their respective neurons’ dipoles. We simulated neuron models with detailed morphologies and distributed passive and voltage-gated ion channels on the soma, axon initial segment, and dendrites (Fig 1A). To induce spiking, we bombarded the dendrites of this active model with background synaptic input, whereas to block spiking in the presence of such synaptic inputs, we set the conductance of voltage-gated sodium channels in the soma and axon initial segment to zero, obtaining a passive model that allowed us to characterize the dipole generated in the absence of firing (Fig 1B). By subtracting the active and passive simulation results and thereafter taking the spike-triggered average of the single-neuron dipole, we estimated the unitary AP response of the electric field (Fig 1C).

The ensemble electric field is equal to the linear summation of those generated by each individual neuron in the brain [24,32]. However, the electric fields generated by individual neurons are not in general linear; sublinear and supralinear interactions among synaptic currents prevent this [33]. Nonetheless, one might hypothesize the contributions of APs to be linear. In this case, the spectrum of the single-neuron dipole, *S* ( *f* )  would be proportional to the energy spectrum of the unitary AP response, *S_ap_*(*f*), satisfying the equation


$$\begin{array}{c} S(\;f\;)=S_{syn}(\;f\;)+\beta S_{ap}(\;f\;), \end{array}$$ (1)

where $S_{syn}(\;f\;)$ is the power spectrum of the synaptic contributions, and *β* is a scaling factor that should be equal to the cell’s firing rate, as we demonstrate below. To test the accuracy of this simplified model, we calculated single-neuron dipoles while varying the firing rate of the neuron by altering the ratio of excitatory to inhibitory input (Fig 1D). We estimated $S_{syn}$ for each EI ratio by considering the passive model in which the sodium channel conductance was set to zero. Meanwhile, $S_{ap}$ was defined as the energy spectrum of the unitary AP response calculated at low firing rates (see Methods). By fitting the power spectrum of the single-neuron dipole at each EI ratio with Eq 1, we estimated the scaling factor *β* and showed that it closely matches the firing rate (Fig 1E and 1F).

**Fig 2:**
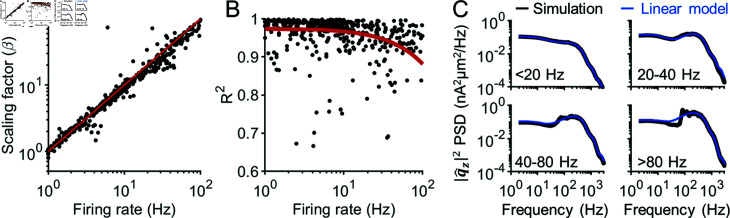
AP contributions to single-neuron dipoles are linear with firing rate. (A) Fitted unitary AP scaling factor (*β*; see Eq 1) plotted against firing rate for 68 neuron models covering the 55 neuron classes identified by Markram et al. [34] (S1 Table). These data points align almost perfectly with the unity line (red line). (B) The $R^{2}$ value obtained from fitting a linear model (Eq 1) to the spectra of the AP dipole response in each simulation. Notice how the line of best fit (red line) shows that Eq 1 gets less accurate at high firing rate. (C) The spectra of the z component of the single-neuron dipoles (black), averaged across all simulations with firing rates in the specified ranges, compared to the spectra predicted from the linear model (blue). For firing rates less than 80 *Hz*, the linear model produces spectra nearly identical to the simulations. At firing rates above 80 *Hz*, there is a slight departure is spectral density around 100 *Hz*. The same results were obtained with the x and y components of the dipole (S1 Fig).

We performed this analysis on biophysical models of 68 representative neuron classes [34] (S1 Table). Across all models, the unitary AP scaling factor *β* closely followed the firing rate (Fig 2A). However, as the firing rate increased, the accuracy of the linear approximation decreased (Fig 2B). This was because the unitary AP responses were less representative of the AP responses occurring at high frequencies. Nonetheless, we found that the spectral profile of the AP-generated signal was nearly identical to that predicted by the linear model up to firing rates of approximately 80 *Hz* (Fig 2C). We concluded that the amplitude of AP responses are captured well by a linear model, but that the precise spectral properties of these responses may be slightly different than those predicted by a fully linear model during sustained high frequency firing above 80 *Hz*. However, considering that the average firing rates of active neurons typically fall below 60*Hz* [35–38], we deemed this simplification of AP signals to be acceptable.

### A linear model for the spectrum of AP electric fields

The above results indicate that the EEG signal can be reasonably approximated as the superposition of a signal generated by synaptic currents and another generated by APs, i.e., the apEEG signal. Furthermore, this apEEG signal can itself be described as a linear system. It follows (see Methods) that the amplitude and spectral features of AP contributions to scalp EEG can be described as


$$\begin{array}{c} |{\hat{\phi }}_{AP}{|}^{2}=\overset{N}{\underset{i=1}{\sum }}{\hat{R}}_{i,i}^{spike}\nu^{⊺}({\text{x}}_{i}){\hat{\text{R}}}_{i,i}^{ap}\nu ({\text{x}}_{i})+\underset{i\neq j}{\sum }{\hat{R}}_{i,j}^{spike}\nu^{⊺}({\text{x}}_{i}){\hat{\text{R}}}_{i,j}^{ap}\nu ({\text{x}}_{j}), \end{array}$$ (2)

where *N* is the total number of neurons in the cortex, ${\text{R}}_{i,i}^{ap}(\tau )$ is the auto-correlation matrix of the unitary AP response for neuron *i*, ${\text{R}}_{i,j}^{ap}(\tau )$ is the cross-correlation matrix for two neurons *i* and *j*, $R_{i,i}^{spike}(\tau )$ is the spike train auto-correlation of neuron *i*, $R_{i,j}^{spike}(\tau )$ is the spike train cross-correlation of neurons *i* and *j*, and ${\hat{\text{R}}}_{i,i}^{ap},{\hat{\text{R}}}_{i,j}^{ap},{\hat{R}}_{i,i}^{spike}$ and ${\hat{R}}_{i,j}^{spike}$ denote their Fourier transforms, respectively. Note that all correlations are unnormalized, e.g., spike train correlations scale with firing rate. Finally, $\nu ({\text{x}}_{i})$ is the “lead field” [32] that transforms the current dipole at a location ${\text{x}}_{i}$ into an electric potential at the scalp.

We estimated ${\text{R}}_{i,j}^{ap}$ from the unitary AP responses generated by all 1035 neuron models from the Blue Brain project [34] using the framework outlined in the foregoing sections. As expected, when the neurons fired APs with zero lag, their dipoles exhibited strong cross-correlations along the apical-basal axes of their respective neurons (S2 Fig). Interestingly, these calculations also revealed significant cross-correlations between the dipoles’ apical-basal component and their azimuthal components (S2 Fig). This observation suggests that even neurons that are not aligned in parallel may still generate coherent electric fields during synchronous firing, thus further boosting the signals generated by populations of AP responses. To compute the final ensemble apEEG spectrum, assumptions need to be made about the spatiotemporal properties of spike synchrony, $R_{i,j}^{spike}(\tau )$, throughout the cortex.

### Magnitude of apEEG depends on dendrite asymmetry

**Fig 3:**
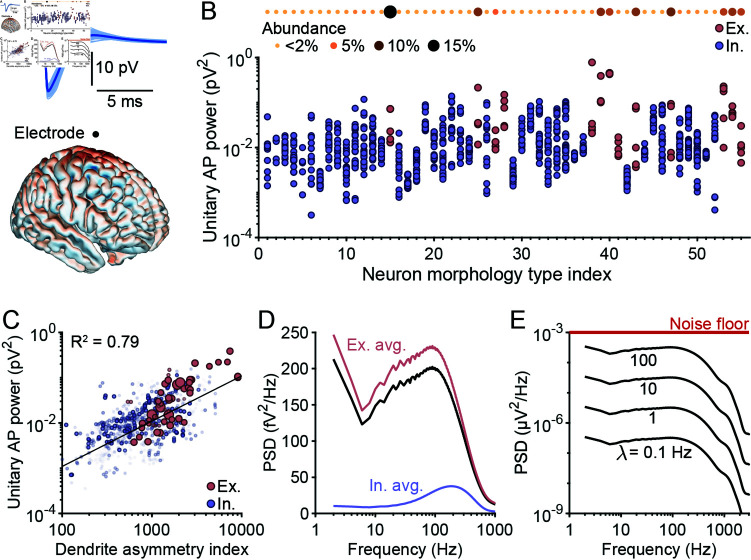
Diverse cell types’ unitary AP contributions to scalp EEG. (A) Top: several AP-aligned single-neuron EEG transients (light blue), along with their average (dark blue), produced by a neuron placed at a random cortical location in the New York Head model (bottom) and measured at the Cz electrode site. (B) The unitary apEEG power, averaged across simulations of all possible neuron locations in the cortex, for each of the 1035 neuron models, split into various morphology classes; a description of each morphology class is provided in S2 Table. Excitatory neurons are shown in red and inhibitory neurons in blue. The relative abundance of each morphology type is shown at the top of the panel. (C) The location-averaged unitary apEEG power of each neuron model plotted against the neuron’s dendrite asymmetry index (Eq 17, see Methods). The size and opacity of each point is directly proportional to the neuron’s relative abundance in the brain. Black line: line of best fit. (D) The expected unitary apEEG spectrum, ${\bar{S}}_{AP}\left(f\right)$, across all neuron models (black), the excitatory neuron models (red), and the inhibitory neuron models (blue). (E) The simulated apEEG spectrum generated by the entire brain firing asynchronously at various frequencies, compared to the noise floor of a typical EEG amplifier [20].

To begin, we investigated the case where neurons fire according to uncorrelated Poisson spike trains. In this case, ${\hat{R}}_{i,j}^{spike}(\;f\;)=0$ and ${\hat{R}}_{i,i}^{spike}(\;f\;)=\lambda_{i}$, although for simplicity we assumed a single average *λ* for all neurons. This simplifies Eq 2 to


$$\begin{array}{lll} |{\hat{\phi }}_{AP}{|}^{2} & =\lambda \overset{N}{\underset{i=1}{\sum }}\nu^{⊺}({\text{x}}_{i}){\hat{\text{R}}}_{i,i}^{ap}\nu ({\text{x}}_{i})\; & \; \end{array}$$ (3)

$$=\lambda N{\bar{S}}_{AP}(f),$$ (4)

where ${\bar{S}}_{AP}\left(f\right)$ is the average spectrum of a unitary apEEG response—a transient in the EEG signal caused by a single AP (Fig 3A). To estimate ${\bar{S}}_{AP}\left(f\right)$, we placed each neuron model at every cortical location in the head model and calculated the unitary apEEG responses (S3 Fig). We then calculated the spectra of these responses and averaged them together, weighting each model’s output by the fraction of cortical neurons it represented (Fig 3B). These simulations produced both an average spectrum and provided information on the contributions of each neuron class to this average spectrum.

Among neuron classes, the average power of the unitary apEEG response varied by almost two orders of magnitude (Fig 3B). Excitatory pyramidal cells tended to generate larger amplitude apEEG signals than inhibitory neurons, as expected [29]. However, certain inhibitory neurons also generated surprisingly large amplitude signals (Fig 3B). Whereas the average excitatory neuron generated a unitary apEEG response with an energy of  ~ 0 . 09 pV^2^, the average inhibitory neuron generated signals of  ~ 0 . 02 pV^2^. Because pyramidal neurons are thought to dominate EEG signals due to their polarized dendrite morphology, we hypothesized that many interneurons have significant asymmetries in their dendritic arbours. To test this, we defined a dendrite asymmetry index (Eq 17; see Methods) and evaluated the predictive power of this measure on apEEG signal strength. Consistent with our hypothesis, the unitary apEEG power for each neuron was strongly correlated with its dendrite asymmetry index (Fig 3C). While in general excitatory neurons exhibited more dendrite asymmetry, many interneuron dendrites displayed equal or greater asymmetry (Fig 3C). This result demonstrates that interneuron spikes can generate large electric fields, commensurate with those of many excitatory neurons. Nevertheless, the average unitary apEEG spectrum, ${\bar{S}}_{AP}\left(f\right)$, was dominated by the spectral features of excitatory neurons as they comprised approximately 85% of all neurons (Fig 3D).

Interestingly, the expected unitary apEEG spectrum revealed both low pass and bandpass properties (Fig 3D). The bandpass property, which is reflected in the peak in the power spectrum around 100 *Hz*, arises from the fast temporal dynamics of the up and downstroke of the AP waveform. The low-pass filtering properties are evident in the low frequency power below 10 *Hz*. This power was disproportionately contributed by certain neuron classes which exhibited significant, slow after-hyperpolarizations that often took tens to hundreds of milliseconds to return to baseline (S4 Fig).

Finally, we examined the ensemble apEEG spectrum (Eq 4). Even with an unrealistically high brain-wide firing rate of 100 *Hz*, the amplitude of the ensemble apEEG signal barely reached the noise floor of high resolution, low noise EEG recordings (Fig 3E). Given the absence of synchrony, these spectra serve as indicators for defining lower bounds on any contributions of APs to scalp EEG. Unsurprisingly, simulated APs were insufficient to produce detectable EEG signals when firing asynchronously.

### Spike synchrony cannot produce high frequency broadband apEEG

**Fig 4:**
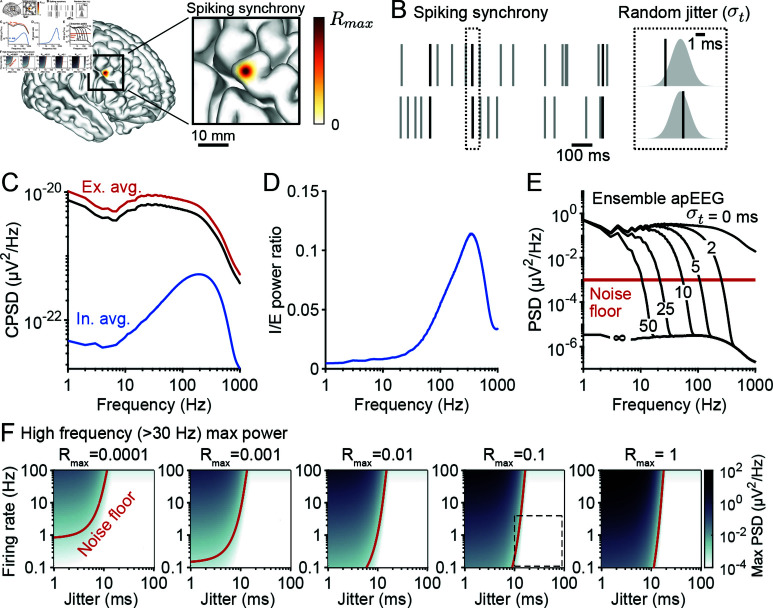
Aperiodic APs cannot generate high frequency EEG signals. (A) Schematic illustration of the local nature of correlated activity in the model. Neighbouring neurons fire spikes with a correlation of $R_{max}$, while neuron pairs that are increasingly separated show gradually decreasing correlation. (B) Schematic illustrating the timescale of correlation. Correlated neurons have a given fraction of their spikes synchronized (left) with a jitter value drawn from a Gaussian distribution of standard deviation $\sigma_{t}$ (right). (C) The average cross-spectral density (CSD) among pairs of excitatory neurons (red), pairs of inhibitory neurons (blue), and all pairs of neurons (black), given the correlation scheme in panels A and B. (D) The ratio between the average CSD for inhibitory and excitatory neuron pairs. (E) Example ensemble apEEG spectra of a brain with an average firing rate of 1 *Hz* and maximal correlation of $R_{max}=0.2$, plotted for various values of $\sigma_{t}$ (blue). Red line: Noise floor of a typical EEG amplifier [20]. (F) Maximal spectral density above 30 *Hz* generated by the model for a whole range of firing rates (*λ*) and jitter values ($\sigma_{t}$), as well as for various maximal correlation values ($R_{max}$). Red line: The boundary delineating spectral density above and below the noise floor of the amplifier. Dotted box: the regime of physiologically realistic parameter values (see Methods).

We next investigated the effects of spike synchrony on apEEG generation, and turned to the full Eq 2. We used a minimal model for spike synchrony based on two general observations:

1. Spike synchrony is strongest among nearby neurons [39–41]. This was implemented in our model by synchronizing the spike timing of neurons depending on their pairwise distance according to $R_{i,j}^{spikes}(\tau )\propto \exp(-d_{i,j}^{2}∕2\sigma_{x}^{2})$, where $d_{i,j}$ is the Euclidean distance between neurons *i* and *j*, and $\sigma_{x}^{2}$ is a parameter that controls the cortical distance over which activity becomes uncorrelated. In accordance with unit recordings in visual cortex [39,40], we set $\sigma_{x}^{2}$ to be 3 mm^2^ (Fig 4A). Although recordings from prefrontal cortex suggest a slightly lower value of around 1 mm^2^ [42], differences in the value of $\sigma_{x}$ at the scale of millimeters did not have meaningful effects on the results that follow (S6 Fig).
2. Even neurons with correlated spiking do not fire at exactly the same time. The timescale of correlation was captured by modelling the spike train cross-correlation as a Gaussian function, whose variance, $\sigma_{t}^{2}$, reflects the jitter in spike times [39–41,43] (Fig 4B).

Together, these two experimental observations give rise to the following equation describing spike synchrony


Ri,jspikes(τ)=λRmax2πσt2 exp ⁡(-di,j2∕2σx2)exp ⁡(-τ2∕2σt2), (5)

where $R_{max}$ represents the noise correlation between neurons [43] and *λ* is the average firing rate of the neurons. According to this model, the dynamics of AP firing and synchrony are both entirely aperiodic, thus allowing us to examine whether apEEG signals can generate aperiodic EEG signals and contribute to the EEG spectral trend.

Under this correlation scheme, we estimated the cross-spectral density among the apEEG responses of all pairs of neurons in the brain, i.e., the second term in Eq 2, using Monte Carlo simulations (see Methods; Fig 4C). By excluding excitatory or inhibitory neurons from these simulations, we could estimate the consequences of spike synchrony in each population separately, which allowed us to test how much apEEG power may be derived from interneuron synchrony versus pyramidal neuron synchrony. These simulations revealed that interneurons can exhibit high coherence in their apEEG signals, especially around  ~ 300*Hz* (Fig 4C). Indeed, the average cross-spectrum among interneurons reached 10% of that among pyramidal neurons (Fig 4D). This suggests that if the rate and/or synchrony of firing (specifically $\lambda R_{max}$) among inhibitory neurons were 10 times higher, they would contribute equally with pyramidal neuron pairs to apEEG power between 250 and 500 *Hz* (Fig 4D). Although the high abundance of pyramidal neurons significantly boosts their contributions to the ensemble EEG signal, we conclude that their specific polar geometry is not strictly necessary for generating coherent AP electric fields.

Considering these coherent fields among excitatory and inhibitory neurons, how large might ensemble apEEG signals get? Could APs contribute to the high frequency plateau seen in EEG spectra? Using our estimates for the average auto-spectra (Fig 3D) and cross-spectra (Fig 4C) of single-neuron apEEG signals, we computed the ensemble apEEG spectrum from Eq 2. As a specific example, Fig 4E shows the spectra calculated for $R_{max}=0.2$ and *λ* = 1 *Hz*. When $\sigma_{t}=0$, spiking occurs with perfect synchrony, producing an apEEG spectrum that is essentially a scaled version of the average cross-spectrum among unitary AP responses. This spectrum indicated that synchronized, aperiodic APs would produce large amplitude, high frequency broadband EEG signals well above the noise floor of typical EEG amplifiers (Fig 4E). On the other hand, when $\sigma_{t}=\infty$, the spectrum was identical to the asynchronous case (Fig 3) and lay far below the noise floor (Fig 4C). For intermediate values, the spectra followed the perfectly synchronous spectrum until a cut-off frequency, determined by $\sigma_{t}$, above which the spectrum dropped down to the asynchronous spectrum (Fig 4C). This indicates that the timescale of correlation, $\sigma_{t}$, is critical in allowing or preventing APs from generating high frequency, broadband EEG signals.

To investigate further, we performed a full sensitivity analysis of model outcomes with respect to the jitter ($\sigma_{t}^{2}$), maximal correlation ($R_{max}$), and firing rate (*λ*). Fig 4D illustrates the maximal spectral density produced at frequencies above 30 *Hz*. The red line indicates where the apEEG crosses the noise floor and the dotted box shows a physiologically reasonable parameter range for *λ*, $\sigma_{t}$, and $R_{max}$ (see Methods: Determining ranges for parameters). This dotted box is almost entirely contained below the noise floor of EEG amplifiers. In other words, realistic firing rates and temporal imprecision in spike synchrony prevent broadband apEEG signals from generating high frequency EEG. We thus conclude it is extremely unlikely that APs contribute to the high frequency plateau observed in EEG spectra.

### Predicted apEEG magnitudes would be too small to shape the spectral trend

The main neural sources of EEG, including broadband EEG, are thought to be synaptic currents [3–7,24,44–46]. However, these scalp potentials are contaminated with broadband external noise, e.g., electromyogram (EMG) [20,22] and amplifier noise [47], as well as narrowband noise, i.e., 50 or 60 *Hz* line noise. To further examine whether APs might constitute a neural source of broadband EEG, we sought to compare our simulation results to EEG data while accounting for other sources of broadband signals.

**Fig 5:**
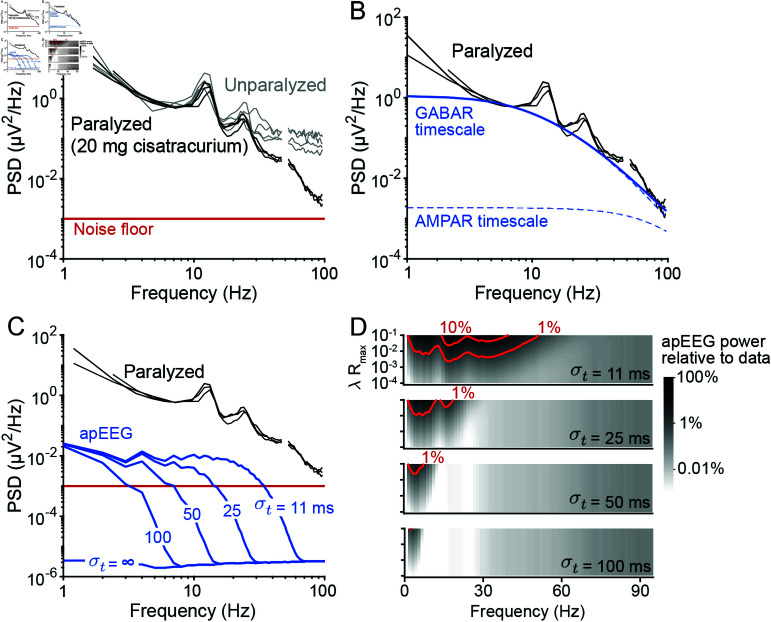
EEG spectral trend can be explained entirely by synaptic timescales. (A) Spectra of EEG signals collected from unparalyzed (grey) and paralyzed (black) subjects by Whitham et al. [20]. Here, the paralyzed spectrum has been noise-floor corrected (see text). (B) The noise-floor corrected spectra of paralyzed individuals, fit with Eq 18 (solid blue; see Methods). The dashed blue lines indicate the contributions of GABA receptor (GABAR) and AMPA receptor (AMPAR) timescales to the fit. Parameter values: $\tau_{I1}=4$ *ms*, $\tau_{I2}=20$ *ms*, $\tau_{E1}=1$ *ms*, $\tau_{E2}=3$ *ms*, $A_{I}=3.6$, and $A_{E}=3.3$. S7 Fig shows that the scaling values $A_{I}$ and $A_{E}$ are consistent with biophysical simulations. (C) Same data as panel B, here plotted alongside the apEEG simulations from Fig 4E. (D) The ratio between the data and simulated apEEG spectra for various $\lambda R_{max}$ values, ranging from $10^{-4}$ to $10^{-1}$ (y-axis of each sub-panel), and $\sigma_{t}$ values, ranging from 11*ms* to 100*ms* (top to bottom). Red contour lines show where the apEEG spectra attain 1% and 10% of the power seen in the data.

This was done be analyzing the EEG data that Whitham et al. [20] collected from subjects during peripheral blockade of nicotinic cholinergic receptors, which causes muscle paralysis and attenuates myogenic artifacts [20,22]. Using the technique from Miller et al. [5], we subtracted the noise floor from the spectra, in order to approximate an EEG spectrum free of the two major known sources of external broadband noise. As expected, these EMG-attenuated, noise floor-corrected spectra no longer exhibited a high-frequency plateau (Fig 5A); this was in agreement with our conclusion that APs cannot produce a high-frequency plateau in EEG spectra.

We next asked how much of the remaining broadband features could be explained by synaptic currents. Past modelling studies have suggested that the EEG spectral trend reflects the timescales of inhibitory and excitatory synaptic currents, with each contributing a “knee” to the spectrum, one at a lower frequency and another at a higher frequency, respectively [3,4]. We therefore attempted to fit the spectrum with the sum of two Lorentzian functions (see Methods). We found that the spectral trend could be almost entirely explained by inhibitory synaptic timescales (Fig 5B), consistent with the profound effect GABA receptor agonists have on the scaling of EEG spectra [3,48]. Excitatory timescales were estimated to contribute modestly at higher frequencies, around 100*Hz*.

Are these estimated synaptic contributions biophysically reasonable? To address this, we extended a previous model of synaptically generated aperiodic EEG [3] to include variable coherence among excitatory and inhibitory currents (S7 Fig). This extension allowed us to examine how different levels of excitatory and inhibitory coherence impact the amplitude and scaling of EEG spectra. We found that the EEG spectral trend from paralyzed subjects could indeed be captured by biophysical simulations of aperiodic synaptic activity (S7 Fig). In our simulations, we reduced the contributions of excitatory timescales relative to inhibitory timescales by constraining the spatial extent of excitatory synchrony. This result could also have been achieved by reducing the amplitude of excitatory synchrony or by perturbing the spatial organization of correlated synapses, as previously shown [3]. We therefore cannot draw definitive conclusions about which mechanism(s) might cause the differences in amplitude between AMPA and GABA mediated timescales in the data, except insofar that these differences are biophysically plausible. Broadly speaking, the simulations and data analysis suggest that the EEG spectral trend can be almost entirely explained by coherent, inhibitory synaptic currents, along with EMG and amplifier noise.

Even if APs do not contribute to the data from Whitham et al. [20], it is possible that APs could contribute under other conditions. By comparing the amplitude of simulated apEEG signals under various parameters to the EMG-attenuated, noise floor-corrected spectra, we determined whether it would be possible for broadband apEEG to ever contribute noticeably to the EEG spectral trend (Fig 5C and 5D). Consistent with our results above, we found that apEEG signals are far to small to produce EEG signals above  ~ 60*Hz* for any physiologically realistic parameter values (Fig 5D). However, we found that aperiodic apEEG signals could potentially contribute small amounts of power at lower frequencies for low jitter values ($\sigma_{t}\sim 10$ *ms*) and high firing rates and/or synchrony levels ($\lambda R_{max}>0.01$). Nevertheless, these parameter values are at the extrema of possible parameter values (see Methods). For most other parameter combinations, particularly with less temporally precise spike synchrony (higher $\sigma_{t}$ values), apEEG could only contribute at most  ~ 1% to the spectral trend observed in the Whitham et al. [20] data. In conjunction with our synaptic timescale analysis, these modelling results suggest that broadband EEG signals are generally produced by synaptic currents, with minimal or no contributions from APs.

### APs can contribute to EEG rhythms in gamma range and above

**Fig 6:**
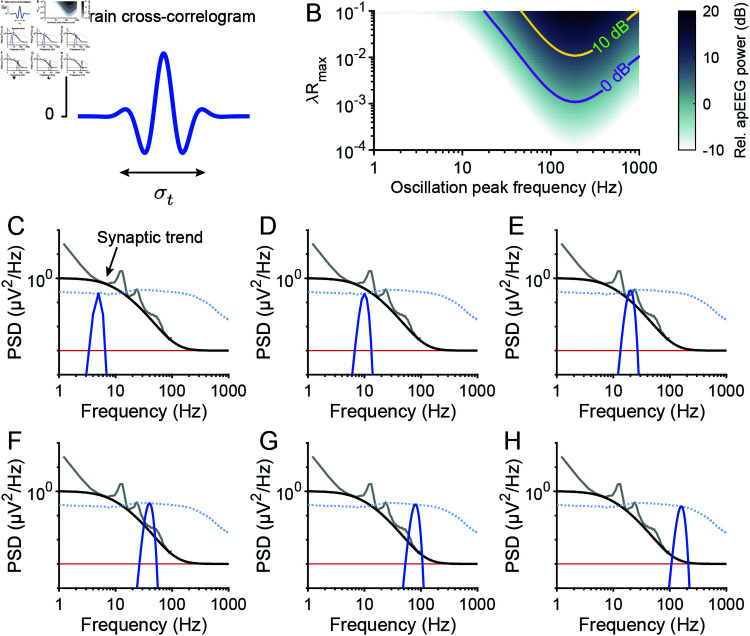
APs can contribute to gamma and higher frequency oscillations. (A) The spike train cross-correlation used to model rhythmic spike synchrony, with $\sigma_{t}=11.3$ *ms* and $f_{0}=40$ *Hz* as an example. (B) Simulated amplitudes of spectral peaks generated by APs synchronized at rhythm frequencies between 1 and 1000 *Hz*. The simulated peak amplitude was defined relative to the power of fitted spectral trend from Fig 5B at the specified oscillation frequency. (C-H) The average spectra of paralyzed patients (grey) and the fitted spectral trend (black). Notice how at higher frequencies, the spectral trend is constrained by the noise floor (red). The simulated spectrum of the apEEG signal (solid blue) generated by rhythmic spike synchrony at $f_{0}=5$ *Hz* (C), 10 *Hz* (D), 20 *Hz* (E), 40 *Hz* (F), 80 *Hz* (G) and 160 *Hz* (H). Dotted blue line: the apEEG spectrum generated by the brain with the same average firing rate and maximal correlation as solid blue lines, but with perfectly synchronized spikes, i.e., $\sigma_{t}=0$ *ms*.

Even if APs do not produce aperiodic EEG signals, it may still be possible that they contribute to EEG rhythms [29]. We therefore used our framework to investigate the consequences of rhythmic synchrony in AP firing activity. We modelled oscillatory synchronization by making the cross-correlation between pairs of neurons a damped sine wave [49,50]. Mathematically, this means that the spike train cross-correlation is now described by the following equation,


Ri,jspikes(τ)=λRmax2πσt2 exp ⁡(-di,j2∕2σx2)exp ⁡(-τ2∕2σt2)cos ⁡(2πτf0), (6)

where $f_{0}$ is the synchronous rhythm frequency. A representative parameterization of this equation is plotted in Fig 6A. Based on this, we investigated the EEG spectra produced by APs when the frequency of synchronous oscillation, $f_{0}$, was systematically varied. The value of $\sigma_{t}$ only altered the sharpness of the oscillations spectral peak, but did not affect its amplitude (S8 Fig); we therefore set $\sigma_{t}$ to maintain a constant peak width in the subsequent simulations.

To evaluate the amplitude of these apEEG oscillations, we computed its power relative to the synaptic trend fit to the data of paralyzed subjects (Fig 5B). In this way, we could compare the modelled amplitude of AP-generated rhythms to a “null” EEG spectrum absent brain rhythms.

When the firing rate or the magnitude of the spiking correlation was too low, APs could not generate any detectable EEG signals (Fig 6B). Interestingly, when the product of the two scaling factors in our model, $\lambda R_{max}$, was above approximately $10^{-3}$, synchronous rhythmic AP firing could generate pronounced spectral peaks in the EEG spectrum, but only if the oscillation frequency was around 200 *Hz*. When the value of $\lambda R_{max}$ was further increased, the regime of oscillation frequencies that produced detectable apEEG signals expanded (Fig 6B).

To better illustrate how these values arose, we plotted one specific example when *λ* = 1 *Hz* and $R_{max}=0.2$. By superimposing the apEEG spectra on the example EEG data, one can see that APs with slower synchronous rhythm frequencies, $f_{0}$, produce apEEG signals below the amplitude of the EEG spectral trend (Fig 6C–6E). However, at higher $f_{0}$, the amplitude of the generated spectral peak increased significantly. The amplitudes of these peaks trace out the spectrum generated by the model when synchrony is entirely aperiodic with zero jitter (Fig 4F–4H). It follows that the amplitude of apEEG rhythms can be predicted by the simplified synchrony model described in the previous section.

For an 80 *Hz* rhythm, the parameter combination $\lambda R_{max}$ needed to be at least  ~ 0 . 03 to produce a signal of equal or greater amplitude than the spectral trend (Fig 6B). This condition could be met, for example, with a maximal correlation value around 0.1 and average firing rates around 0.3 *Hz*, which are within the range of values measured experimentally (see Methods: Determining ranges for parameter values). In contrast, no reasonable set of parameters would allow APs to contribute significantly to EEG signals of lower frequency rhythms. We conclude that lower frequency EEG rhythms likely reflect purely synaptic activity, whereas rhythmic EEG signals in the gamma range or higher may contain significant contributions from APs.

### APs explain EEG oscillations above 250 Hz

**Fig 7:**
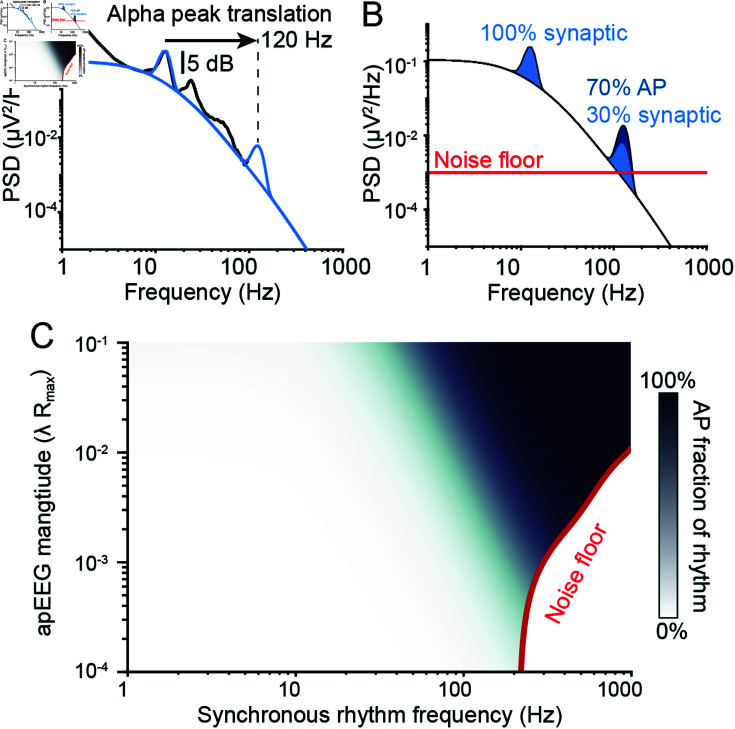
APs can explain high frequency EEG oscillations. (A) Scheme for estimating upper bound on synaptic contributions to spectral peaks at high frequencies. See text for details. (B) Two spectral peaks reflecting the sum of the synaptic component, i.e., panel A, and the apEEG component, shown in Fig 6. APs contribute negligibly to the alpha peak, but are estimated to contribute  ~ 70% to the 120*Hz* peak under these specific model parameters. (B) The fractions that APs are estimated to contribute to spectral peaks at various frequencies, coded in opacity (see color bar), as a function of parameter combination $\lambda R_{max}$. Red line: contour below which the combined synaptic-AP spectral peak does not overcome the noise floor.

Given an EEG oscillation, how much of it is due to synaptic currents versus APs? Even under the most extreme parameter values, our simulations indicate that APs cannot contribute significantly to EEG oscillations at low frequencies (Fig 6C–6H). We used this result to estimate lower bounds on the contributions of APs to higher frequency oscillations. Specifically, we approximated the contributions of synaptic currents at higher frequencies by translating the alpha peak—which we assumed to be generated purely by synaptic currents—along the fitted spectral trend towards the new rhythm frequency (Fig 7A). This procedure has the following mechanistic interpretation. Because of the gating kinetics of AMPA and GABA receptors, synaptic contributions to EEG reflect a low-pass filtered version of the oscillations in neural spiking. Thus, by holding the amplitude of the spectral peak constant relative to the synaptic filter’s frequency response (i.e., the spectral trend), one is assuming that the underlying oscillation only differs in its frequency. In reality, EEG oscillations are known to attenuate for other reasons as well, such as the decrease in spatial synchrony with increasing frequency [51,52]. We therefore took our translated peak as an upper bound for high frequency synaptic contributions.

We combined these surrogate, synaptic peaks with simulated apEEG peaks and estimated the fraction of the ensemble peak contributed by APs (Fig 7B). Due to the upper limit on synaptic contributions, this approach provides a lower bound for AP contributions. This analysis indicated that EEG oscillation faster than 250*Hz* must be due to APs, because any synaptic component would be too strongly attenuated to overcome the noise floor. This can be seen from the fact that above the noise floor cutoff in Fig 7C, any oscillation above 250*Hz* would be predominantly AP generated. At the other extreme, oscillations slower than  ~ 30*Hz* contained minimal apEEG content, no matter how we parameterized the apEEG simulations (Fig 7C), consistent with our results above. For oscillations at intermediate frequencies, the contribution of APs would depend on the precise value of the parameter combination $\lambda R_{max}$ which is unknown in practice. Decreasing the spatial extent of AP synchrony constrained the parameter regime where APs could generate detectable EEG, but still indicated that APs are alone capable of generating detectable EEG oscillations above 250*Hz* (S9 Fig). Altogether, these simulations predict that AP-related currents are responsible for EEG oscillations faster than 250*Hz*, and potentially even those as slow as 80*Hz* depending on yet uncharacterized parameters of apEEG generation.

## Discussion

We have shown using biophysical simulations that asynchronous spiking activity cannot cross the noise floor of EEG amplifiers in our models and that, while synchronous aperiodic spiking can generate detectable EEG signals, these signals are dwarfed by the contributions of synaptic currents. On the other hand, we found that rhythmic spiking activity can potentially generate significant EEG oscillations depending on the frequency of such rhythmicity. Together, our results provide quantitative insights into the neural basis of EEG and have direct practical implications for interpreting EEG spectra.

### Interpreting and analyzing EEG spectra

To detrend or not to detrend EEG spectra depends on the mechanisms underlying the changes in the spectral trend [3]. Our results therefore provide important theoretical guidance for spectral detrending. First, our modelling suggests that high frequency broadband EEG signals are unlikely neural in origin, in constrast to more invasive techniques where APs are thought to contribute. This point highlights that detrending requires different methodologies at high and low frequencies. While additive noise at high frequencies can be corrected through subtractive detrending [5], synaptic timescales should be corrected divisively [3]. Notably, “whitening” EEG spectra typically involves subtracting the log slope of the spectrum [1,53], which is a divisive operation. Our results suggest that this process will overestimate changes in higher frequency EEG oscillations, particularly above 30*Hz*.

**Fig 8:**
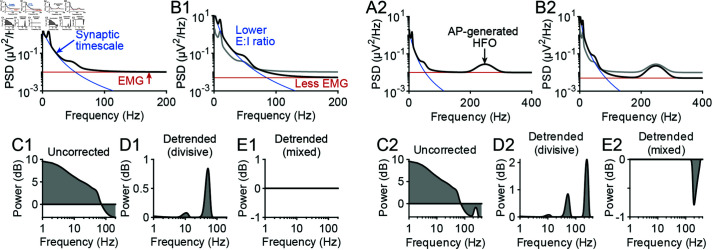
A toy model illustrating the implications of this study’s results on spectral detrending. (A1) EEG spectrum (black) modelled with (i) Gaussian functions at 10 and 40 *Hz*, a constant offset and 1/f noise at low frequencies, all filtered by synaptic timescales (the synaptic timescales fitted in Fig 5 were used), plus (ii) additive EMG noise. The equation describing the spectrum was $P(\;f\;)=(1+\alpha (\;f\;)+\gamma (\;f\;)+1∕f^{2})P_{syn}+noise$, where *α* and *γ* represent the Gaussian peaks. The spectral density from synaptic timescales (blue) and additive noise (red) are overlaid on the simulated spectrum. (B1) Same as in A1, but the EMG noise has been decreased by 50% and the E:I ratio was decreased  ~ 2 . 5 fold. The spectrum from panel A1 is shown in grey for comparison. The amplitude of the the Gaussian functions were not changed. (C1) Power of spectrum after parameter modifications relative to before (i.e., black vs gray lines in B1). Differences in the spectral trend were not corrected for. (D1) The same as in C1, but with the spectral trend, defined as $P_{syn}+noise$, removed divisively from the spectra. Notice how this analysis artifactually displays an increase in the alpha and especially the gamma peak, even though these components of the spectrum were not changed. (E1) The same as in C1, but with spectra detrended using a mixed approach. Here, the EMG noise was subtracted prior to detrending divisively with the synaptic timescales, producing correctly no changes in rhythmic power. (A2-E2) The same as in A1-E2, respectively, but with the inclusion of a high frequency oscillation (HFO) at 250*Hz* generated by APs using the equation $P(\;f\;)=(1+\alpha (\;f\;)+\gamma (\;f\;)+1∕f^{2})P_{syn}+HFO(\;f\;)+noise$.

We illustrate this point using a toy model in Fig 8. Suppose two EEG spectra are being compared, one of which reflects a lower excitatory to inhibitory (E:I) ratio and less electromyogram (EMG) (Fig 8A1 and Fig 8B1). Due to the difference in E:I ratio, the spectra need to be detrended of synaptic timescales prior to comparison, as described previously [3,4] (Fig 8C1). However, in cases where the high frequency plateau is also changing, for example due to differences in muscle tone, the high frequency plateau needs to be subtracted first. Otherwise, peak estimates will be nonuniformly biased (Fig 8D1 and Fig 8E1).

Importantly, correcting for synaptic timescales assumes that spectral peaks are generated by synaptic currents. Our results suggest that this is may not be the case for high frequency oscillations. In this case, correcting for synaptic timescales when analyzing high frequency oscillations would lead to incorrect conclusions (Fig 8A2–Fig 8E2). Our results thus strongly indicate that high frequency oscillations should be analyzed separately from lower frequency oscillations. Our simulations provide a reasonable frequency range where oscillations can be generated by AP activity and therefore suggest principled cutoff frequencies for performing different spectral trend analyses. Nonetheless, lower bounds for this frequency cutoff depended on parameters for which we still lack brain-wide estimates, including levels of spiking synchrony and average firing rates. Spectral analysis of EEG would thus benefit from future experimental work investigating these parameters in more detail as the necessary techniques for collecting large scale, simultaneous spiking data continue to improve [54]. Furthermore, future work applying our modelling framework to determine AP contributions to intracranial EEG could be useful for extending our conclusions to patients with epilepsy who exhibit high frequency oscillations [55–57].

### Neural basis of EEG

Past work has found that apEEG signals can in theory produce low frequency EEG oscillations [29], contrary to our findings. In this previous study, the relative contributions of APs and synaptic currents to the single-neuron dipole were investigated, concluding that APs contribute up to 20% of the single-neuron dipole signal. Our simulations indicated that the average unitary apEEG response is approximately 0 . 08 pV^2^, whereas the average single-neuron EEG power generated in our passive simulations was 0 . 09 pV^2^. Thus, at the single-neuron level, our results agree with this previous study, assuming an average firing rate of approximately 0 . 25*Hz*. However, this ratio would only persist in the ensemble EEG if the synaptic and AP components were similarly coherent. Our findings indicate that, even at upper parameter bounds, APs and their after-hyperpolarizations can contribute only minimally to lower frequency EEG rhythms. Our results thus demonstrate that the relative contributions of APs and synaptic currents to single-neuron signals do not carry over to ensemble EEG signals. Nonetheless, our results do indeed suggest that at higher frequencies, APs can account for significant fractions of EEG rhythms, potentially forming the sole neural basis of these rhythms above 250*Hz*.

Our work also has implications for understanding the cellular basis of EEG signals. In particular, EEG signals are typically thought to be generated by pyramidal neurons [24,58]. However, despite the prototypical morphology of an inhibitory neuron as a stellate cell, with a closed field structure that would prevent them from generating EEG signals [59], we found that the slight asymmetries in interneuron morphologies allowed them to generate unitary apEEG signals only four-fold smaller than excitatory neurons. This result is broadly in line with those of Tenke et al. [60], who found that asymmetries in stellate cells allow them to exhibit open field configurations and generate significant current source densities.

Moreover, we found that almost the entirety of the apEEG signal amplitude is conferred by spiking synchrony. While the parallel morphologies of pyramidal neurons allowed their apEEG signals to cohere more strongly, even interneurons exhibited the ability to generate coherent electric fields, especially between 250 and 500 Hz. While differences in spiking dynamics are not well quantified in vivo, interneurons are typically thought to fire faster and more synchronously than excitatory neurons [61]—especially parvalbumin-expressing (PV) interneurons which account for  ~ 30% of all interneurons. It may therefore be possible that interneurons contribute significantly to high frequency apEEG signals. Coincidentally, the frequency range where inhibitory neurons generate the strongest ensemble apEEG signals (250–500 Hz) is also the frequency range where our model suggested EEG rhythms could be predominantly AP-driven. In future work, it would be interesting to quantify precisely the degree to which experimentally determined differences in firing rate and spike synchrony compensate for the weaker unitary AP responses and lower abundance of inhibitory neurons. Combined with our other results, these insights may inform what cell types are predominately responsible for high frequency EEG rhythms.

### Modelling assumptions and limitations

In modelling the apEEG signal, we assumed that the AP component of the EEG is independent of the synaptically-generated EEG signal; that is, in Eq 10 we assumed a null cross-spectrum between electric fields generated by APs and postsynaptic potentials (PSPs). The AP-PSP cross-spectrum could be estimated from simulations of neural circuits with morphologically detailed neuron models. However, the results would depend strongly on the precise network topology and sub-cellular targeting of synaptic connections which remain only vaguely constrained by experiments. As more data emerge on cellular and sub-cellular connectivity patterns, it could be worthwhile to examine this assumption in more detail.

Nonetheless, we do not expect that including the cross-spectrum would qualitatively change our study’s conclusions. Firstly, in an aperiodic regime, the majority of power from subthreshold fluctuation will be unrelated to the timing of spikes due to the cancellation between excitatory and inhibitory currents [62] (Fig 1). In this case, a null cross spectrum is a reasonable approximation and we therefore expect our results pertaining to broadband apEEG to be robust. During synchronous oscillations, both synaptic and AP events are strongly entrained, and their correlation could either increase or decrease the power of EEG oscillations depending on the nature of the cross-spectrum. Regardless, the amplitude of this modification is bounded above by $|{\hat{\phi }}_{syn\times AP}|\leq \sqrt{|{\hat{\phi }}_{syn}{|}^{2}|{\hat{\phi }}_{AP}{|}^{2}}$; in other words, the low pass filtering of synaptic kinetics would prevent the cross-spectrum from having a large impact on high frequency EEG power. Consequently, quantifying the cross-spectrum would likely not change our conclusions about the neural sources of high frequency oscillations.

Finally, recent work has suggested that APs propagating along axons generate dipoles that contribute to LFP recordings [63]. On the other hand, it has been argued that due to the random orientations of axonal termination segments, these signals would not contribute to EEG [29]. In the current study, we only considered back-propagating APs. However, a similar theoretical framework could be used to investigate the electric fields generated by forward propagating APs in more detail. Including these contributions could increase the amplitude of the unitary AP response, potentially expanding the parameter range that permits AP-generated EEG oscillation. On the other hand, including forward propagating APs would not change our finding that jitter in spike timing prohibits high frequency aperiodic apEEG signals, and therefore our conclusions pertaining to the spectral trend would likely be unaffected.

### Conclusion

Based on our findings, we conclude that APs are unlikely to contribute to the EEG spectral trend, and that this trend can be explained entirely by synaptic timescales, electromyogram contamination, and amplifier noise. However, we also conclude that APs can produce narrowband EEG power at high frequencies, with potentially significant contributions from interneurons. If true, these model predictions suggest that high frequency oscillations and low frequency oscillations interact with the spectral trend differently. While low frequency oscillations require detrending of synaptic timescales, applying this analysis to EEG oscillations generated by APs would produce incorrect results. Altogether, this work indicates that the interactions between spectral peaks and the spectral trend is frequency dependent, further highlighting that the EEG spectral trend is not a singular phenomenon and should not be removed as a single parameterized function when higher frequencies are included.

## Methods

### Mathematical framework

We will derive an equation for the contributions of APs to scalp EEG assuming that the EEG signal can be treated as a linear superposition of a signal generated by APs and a signal generated by synaptic currents, an assumption we validate in Figs 1 and 2. In general, the potential between two electrodes can be calculated from their lead field [32], *ν* ( ) , which describes the sensitivity of the measured potential with respect to a unit dipole vector at the spatial point . Using this formalism, we can write the potential generated by *N* neurons as


$$\begin{array}{c} \phi (t)=\overset{N}{\underset{i=1}{\sum }}\nu^{⊺}({\text{x}}_{i}){\text{q}}_{i}(t), \end{array}$$ (7)

where ${\text{q}}_{i}$ is the single-neuron dipole of neuron *i*, located at coordinate ${\text{x}}_{i}$ in the brain. This equation leads to the following power spectrum for the ensemble signal


$$\begin{array}{c} |\hat{\phi }{|}^{2}=\underset{i,j}{\sum }\nu^{⊺}({\text{x}}_{i}){\hat{\text{R}}}_{i,j}\nu ({\text{x}}_{j}), \end{array}$$ (8)

where ${\text{R}}_{i,j}(\tau )$ is the cross-correlation matrix of the single-neuron dipoles for neurons *i* and *j*, and ${\hat{\text{R}}}_{i,j}$ denote their Fourier transforms.

We now make use of the superposition assumption by decomposing ${\text{q}}_{i}$ into its synaptic and AP component


$$\begin{array}{c} {\text{q}}_{i}(t)={\text{q}}_{syn(i)}+{\text{q}}_{AP(i)}, \end{array}$$ (9)

where the vector ${\text{q}}_{syn(i)}$ is the synaptic component of the single-neuron dipole for neuron *i*, and ${\text{q}}_{AP(i)}$ is the AP component of the dipole of neuron *i*. This decomposition allows us to rewrite the ensemble EEG power spectrum as


$$\begin{array}{c} |\hat{\phi }{|}^{2}=|{\hat{\phi }}_{AP}{|}^{2}+|{\hat{\phi }}_{syn}{|}^{2}+2Re({\hat{\phi }}_{AP\times syn}), \end{array}$$ (10)

where $|\hat{\phi_{AP}}{|}^{2}$ is the apEEG power spectrum, $|\hat{\phi_{syn}}{|}^{2}$ is the synaptic EEG power spectrum, and ${\hat{\phi }}_{AP\times syn}$ is the cross-spectrum between these two signals. In the present study, we will assume that the AP-synapse cross spectrum is negligible (see Discussion). Consequently, the contributions of APs to scalp EEG will be characterized by the amplitude and spectral properties of the term $|{\hat{\phi }}_{AP}{|}^{2}$.

The contributions of APs to scalp EEG can therefore be written, in parallel to Eq 8, as


$$\begin{array}{c} |{\hat{\phi }}_{AP}{|}^{2}=\underset{i,j}{\sum }\nu^{⊺}({\text{x}}_{i}){\hat{\text{R}}}_{i,j}^{AP}\nu ({\text{x}}_{j}), \end{array}$$ (11)

where ${\text{R}}_{i,j}^{AP}(\tau )$ is the cross-correlation matrix between ${\text{q}}_{AP(i)}$ and ${\text{q}}_{AP(j)}$, i.e., the cross-correlation for specifically the AP components of the single-neuron dipoles of neurons *i* and *j*, and ${\hat{\text{R}}}_{i,j}$ denote their Fourier transforms. To arrive at Eq 2, we make use of the results in Figs 1 and 2 that the AP-component of the single-neuron dipole can be described as an impulse response function—that is, a spike train driving unitary AP responses. Mathematically, this means ${\text{q}}_{AP(i)}=({\text{q}}_{ap(i)}\;*\;w_{i})(t)$, where $w_{i}$ is the spike train and ${\text{q}}_{ap(i)}$ is the unitary AP response of neuron *i*. The cross-correlation between ${\text{q}}_{AP(i)}$ and ${\text{q}}_{AP(j)}$ is thus the cross-correlation between their unitary AP responses, ${\text{R}}_{i,j}^{ap}$, convolved with the cross-correlation between their spike trains, $R_{i,j}^{spike}$. After taking the Fourier transforms, we therefore get


$$\begin{array}{lll} {\hat{\text{R}}}_{i,j}^{AP}={\hat{R}}_{i,j}^{spike}{\hat{\text{R}}}_{i,j}^{ap}. & \; & \; \end{array}$$ (12)

Using our above definition for the contributions of APs to scalp EEG, we arrive at the equation we will attempt to estimate throughout this paper,


$$\begin{array}{ll} |{\hat{\phi }}_{AP}{|}^{2} & =\underset{i,j}{\sum }{\hat{R}}_{i,j}^{spike}\nu^{⊺}({\text{x}}_{i}){\hat{\text{R}}}_{i,j}^{ap}\nu ({\text{x}}_{j})\; \end{array}$$ (13)

$$=\sum_{i}R_{i,i}^{spike}v^{T}\left(x_{i}\right)R_{i,i}^{ap}v^{T}\left(x_{i}\right)+\sum_{i\neq j}R_{i,i}^{spike}v^{T}\left(x_{i}\right)R_{i,i}^{ap}v\left(x_{i}\right).$$ (14)

### Biophysical simulations of unitary AP responses

To calculate the unitary AP responses of various neuron types, we simulated 1034 biophysical neuron models originally developed by the Blue Brain Project [34]. These models have detailed morphological reconstructions of the dendritic arbours and 13 voltage-dependent channels distributed throughout the axonal, somatic, and dendritic segments. For the present work, background synaptic input was added to the model to drive AP firing by distributing 1 excitatory synapse and 0.15 inhibitory synapses per µ*m* of dendrite. The average firing rate of all synapses was set to 1.75 *Hz* and the ratio of excitation to inhibition, defined as the ratio of mean activation rates between excitatory and inhibitory synapses, was tuned for each neuron model to bring the firing rate below 40 Hz but above 0 Hz. This ensured that APs occurred sparsely enough that the electric fields they generated were independent of one another.

Models were simulated using the python package LFPy [64], built on top of the NEURON simulation environment [65], and the single-neuron dipole generated at each time point was calculated using the totality of the current in the dendritic and somatic compartments, as described by Næss et al.[30]. These simulations were then repeated with spiking abolished by setting the somatic sodium channel conductances to zero. The difference between the single-neuron dipoles was then computed and taken to approximate the AP component of the dipole. To calculate the unitary AP response, APs were identified in the somatic compartment of the spiking neuron models using MATLAB’s findpeaks algorithm with a minimum peak height set to 0 *mV*. The spike-triggered average of the AP component of the dipole was then computed for each neuron model. Every model was simulated sufficiently long to obtain at least 10 spikes for averaging.

To simulate EEG signals, we used the lead field from the New York Head model [66]. The New York Head model is an average head model based on the MRI of 152 individuals and has the lead field calculated at approximately 75,000 cortical mesh points. All the simulations results here are based on the potential at the Cz electrode site measured against the common average reference. Note that when a neuron morphology was placed at a source location in the cortex, it was embedded in the local coordinates. In other words, the apical-basal axis of the neuron morphology was aligned perpendicular to the cortical surface.

### Monte Carlo simulations for apEEG spectrum with spike synchrony

Evaluating the second term in Eq 2 required computing the cross spectra for all pairwise combinations of cortex coordinates and neuron models ($>10^{15}$ unique pairings), making this step intractable. We therefore used a Monte Carlo sampling approach [67]. Because spike synchrony was assumed to be local in nature with


$$\begin{array}{c} {\hat{R}}_{i,j}^{spike}(\;f\;)=ĥ(\;f\;)\exp(-d_{i,j}^{2}∕2\sigma_{r}^{2}), \end{array}$$ (15)

for a spike train cross correlogram of the form *h* ( *t* ) , we could rewrite the second term in Eq 2 as follows


$$=\underset{i\neq j}{\sum }R_{i,j}^{spike}v^{T}\left(x_{i}\right)R_{i,j}^{ap}v\left(x_{i}\right)=h\left(f\right)\int_{r\in R+}e^{-r^{2}/2\alpha_{r}^{2}}{\bar{S}}_{x,y}\left(r\right)dN\left(r\right).$$ (16)

Note the assumption that *h* is independent of space. To calculate this equation, we needed to calculate *dN* ( *r* )  and ${\bar{S}}_{x,y}\left(r\right)$.

The density *dN* ( *r* )  reflects the number of neuron pairs in the cortex separated by a distance *r*, e.g., of all pairs of neurons in the cortex, how many are separated by 5*mm*? We estimated this directly from the anatomical data of the New York head model as described previously [3]. Briefly, we placed a ball of radius *r* centered at some coordinate in the cortex and calculated the total surface area of the cortex within this ball. Assuming a uniform distribution of 16 billion neurons across the cortical surface area [68], we determined the total number of neuron within a radius *r* of this point. We then repeated this procedure with the ball centered at 10000 different points in the cortex and for balls of radii ranging from 0 to 200 *mm*. By averaging across the different center points, we estimated the average number of neurons that exist within *r* *mm* of any point in the cortex (S5 Fig). The derivative of neuron count with respect to *r* gives the value *dN* ( *r* ) .

The spectrum ${\bar{S}}_{x,y}\left(r\right)$ is the expected cross-spectrum between single-neuron EEG signals generated by cells separated by a distance *r* and was estimated using Monte Carlo sampling. First, two neuron models were selected proportionally to the estimated abundance of each morphology type and placed at the same random location, sampled uniformly from the entire cortex. Then, one neuron was displaced in the cortex by a random distance chosen with a probability defined by the distribution *dN* ( *r* ) . From this setup, the unitary apEEG responses for the two neurons at their respective locations were calculated. The cross-spectrum between these two unitary apEEG responses was then included in the estimate of Eq 16. This procedure was repeated approximately 44 million times, which was determined when Chebyshev’s inequality [67,69] indicated there was less than 1% probability that our estimate was off by an absolute error of $\delta_{abs}=4\times 10^{-25}$ μV^2^/Hz, thus corresponding to an error in the ensemble EEG of equal magnitude to the noise floor of typical EEG amplifiers, i.e., approximately $10^{-3}$ μV^2^/Hz [20].

### Dendrite asymmetry index

Each dendritic arbour was defined by *N* truncated cones with volumes $V_{i}$ and midpoints ${\text{x}}_{i}$, for *i* ∈ { 1 , 2 , . . . *N* } . The dendrite asymmetry index was then defined as


$$\begin{array}{c} AI=\left|\overset{N}{\underset{i=1}{\sum }}V_{i}{\text{x}}_{i}⊘\sqrt{\frac{1}{N-1}\overset{N}{\underset{i=1}{\sum }}{\text{x}}_{i}⊙{\text{x}}_{i}}\right|, \end{array}$$ (17)

where  |⋅| denotes the Euclidean norm, while  ⊘  and  ⊙  denote element-wise division and multiplication, respectively. The calculation of this index is illustrated in S10 Fig. Conceptually, this equation measures how far the weighted centroid of the dendrites is from the origin, normalized in a sense by the span of the dendritic tree. We found that normalization by the actual span of the dendrites over-penalized cells with long apical dendrites. Conversely, without any normalization, cells with large dendritic spans could have relatively symmetric dendrites but large absolute asymmetries, causing their apEEG signals to be overestimated. We found that normalizing by the standard deviation balanced these two extremes well.

### Determining ranges for parameter values

#### Magnitude and timescale of correlation.

Co-tuned neurons in area MT exhibit correlations of approximately $R_{max}=0.2$ with a timescale estimated to be around $\sigma_{t}=11.3$ *ms* [43]. Unless otherise stated, we used these values in our example simulations. For two reasons, these parameter values likely represent upper bounds for brain-wide spike train correlations. First, neurons that are not co-tuned exhibit less correlated activity [70], meaning that the average spike synchrony among all neighbouring neurons is likely lower than that between co-tuned neurons. Consistent with this, a survey of studies across different experimental paradigms and cortical areas found correlation values ranging between 0.05 and 0.25 [41]. In area MT, the timescale of correlation was found to be around 10 ms [43], whereas studies in V4 indicate timescales of tens or hundreds of milliseconds [40,71]. We therefore took a liberal range of 10 to 100 *ms* as an acceptable range for $\sigma_{t}$.

#### Mean firing rate.

Because activity is sparse in the cortex, most neurons are silent at any particular moment; this silent fraction has been estimated to be up to 90% of all cells [72]. As a consequence, the average firing rate across the entire brain is significantly lower than would be expected from measurements of only responsive neurons, with estimates averaging around 0.1 to 2 *Hz* [72–74].

### EEG data and spectral trend fitting

The experimental data shown in Fig 5, Fig 6, and Fig 7 were extracted directly from the figures in Whitham et al. [20], and were fit with the equation


$$\begin{array}{c} S(\;f\;)=\frac{A_{I}{\left(\tau_{I1}-\tau_{I2}\right)}^{2}}{\left(1+{\left(2\pi f\tau_{I1}\right)}^{2})(1+{\left(2\pi f\tau_{I2}\right)}^{2}\right)}+\frac{A_{E}{\left(\tau_{E1}-\tau_{E2}\right)}^{2}}{\left(1+{\left(2\pi f\tau_{E1}\right)}^{2})(1+{\left(2\pi f\tau_{E2}\right)}^{2}\right)}, \end{array}$$ (18)

where $\tau_{I1}$ and $\tau_{I2}$ are the rise and decay time constants associated with inhibitory synaptic responses and $\tau_{E1}$ and $\tau_{E2}$ are the rise and decay time constants associated with excitatory synaptic responses, while $A_{I}$ and $A_{E}$ govern the relative contribution of inhibitory and excitatory currents to the EEG spectrum. This equation was adapted from Eq 6 in Brake et al. [3], except that here the high frequency plateau was explicitly decomposed into excitatory synaptic contributions and the noise floor, which was empirically determined to be $\sim 10^{-3}$ μV^2^/Hz [20] and subtracted from the spectrum prior to fitting. The parameters $\tau_{I1}$ and $\tau_{I2}$ were taken to be the same as in Brake et al. [3], while the exact values for $\tau_{E1}$ and $\tau_{E2}$ did not have a significant impact on our conclusions provided they were chosen to be faster than the GABA receptor timescales.

## Supporting information

S1 Fig Scaling of the x and y components of the unitary AP spectrum. (**A–C**) Same as Fig 2, but for the *x* component of the single-neuron dipole. (**D-F**) Same as Fig 2, but for the *y* component of the single-neuron dipole.
(TIF)

S2 Fig Cross-correlation among unitary AP responses. Solid black line indicates average across all pairs of 1035 neuron models, weighted by the relative abundance of the pairing (Fig 3B). Shading reflects 95% confidence interval of the mean.
(TIF)

S3 Fig Example unitary apEEG responses from five neuron models placed at six cortical locations. The source locations are labeled on the brain template at the top of the figure. The lines extending from each labeled location indicate the direction of the respective normal vector, which were used to orient the apical-basal axes of the neurons. The morpohlogies of the five neuron models are shown below the cortical template and are labeled using the syntax from Markram et al. [34]. From left to right, these models represent a layer 6 pyramidal neuron, a layer 2/3 pyramidal neuron, a layer 4 basket cell, a layer 4 chandelier cell, and a layer 5 thick-tufted pyramidal neuron. Below each neuron morphology is shown the x (blue), y (red), and z (orange) components of the single-neuron dipole in response to an AP. Finally, at the bottom of the figure is shown the unitary apEEG responses produced at the Cz electrode when each neuron model is placed at each of the six indicated cortical locations. Note that amplitude of the apEEG signal depends on the overall size of the neuron and the distance from the neuron to the electrode.
(TIF)

S4 Fig Low frequency apEEG power generated by afterpotentials. (**A**) The slope of the unitary AP spectrum for every neuron model, calculated between 1-10 Hz. A neuron (ID: L4_BP_bAC217_1) with a particularly negative slope is indicated in blue. (**B**) Power spectrum of the unitary AP spectrum for the neuron indicated in panel A, with 1/f trend fitted at low frequencies (dashed black line). (**C**) Top: z component of the single-neuron dipole of the neuron indicated in panel A (black) and with somatic and axonal sodium channels removed to generate a passive model (grey). Bottom: Difference between the active and passive neuron models. Note that the spikes have been truncated at  ± 0 . 5 *nA*�*m*. After each spike, the active model’s dipole takes hundreds of milliseconds to reconverge to the passive model. The same phenomena were observed in the dipoles x and y components.
(TIF)

S5 Fig Distribution of neuron pairs with respect to pairwise distance. (**A**) Surface area (SA) of the cortex from New York head model enclosed within balls of increasing radii, with the origin of the ball placed at 10,000 cortical locations. Black dots indicate the discrete ball radii for which the surface area was calculated. Shading reflects standard deviation across the 10,000 starting points. (**B**) The derivative of the surface area with respect to radius (black), scaled to obtain the density of neuron pairs for each pairwise distance (see Methods). The red curve illustrates the coupling kernel, as in Fig 4A. The vast majority of neuron pairs are separated by more than 10 *mm* and are therefore not correlated in the model.
(TIF)

S6 FigSensitivity of aperiodic apEEG to $\sigma_{\text{x}}$ .(**A**) Same as in Fig 4F, but for $\sigma_{x}^{2}=1$ mm^2^. (**B**) Same as in Fig 4F. (**C**) Same as in Fig 4F, but for $\sigma_{x}^{2}=5$ mm^2^. (**D**) Left: Same as in Fig 5D, but for $\sigma_{x}^{2}=1$ mm^2^. Middle: Same as in Fig 5. Right: Same as in Fig 5D, but for $\sigma_{x}^{2}=5$ mm^2^ (left).(TIF)

S7 FigValidation that AMPA and GABA receptor timescales can scale independently.(**A**) Illustration from Brake et al. [3] of the minimal model for dipole coherence between two neurons. Briefly, synapses on the two neurons are activated with correlated Poisson processes, with correlation strengths determined by the angular distance between the synapses. See Brake et al. [3] for more details. (**B**) Only GABA receptor activation has been correlated. Left: the average power spectral density (PSD) and cross spectral density (CSD) for the two single-neuron EEGs. Middle: The PSD has been fit with the sum of two Lorentzian functions (Eq 18) (green). The GABA receptor (blue) and AMPA receptor (red) related timescales are also shown. Right: The CSD is entirely fit with the GABA receptor timescale from the middle panel. In other words, the cross spectrum is entirely determined by the timescale of the correlated GABA receptors. (**C**) The PSD of the summed single-neuron EEGs from the two neurons when the synapses were correlated and independent. Both PSDs were fit with Eq 18 (black lines) using the same $\tau_{I1}$, $\tau_{I2}$, $\tau_{E1}$, and $\tau_{E2}$ as the PSD in panel B. As expected from the CSD, the difference between the correlated and independent ensemble EEG was captured by increasing the scaling of the GABA receptor timescales. (**D**) Same as panel B, but here only AMPA receptor activation has been correlated. Now the CSD (right) is captured entirely by the timescale of the AMPA receptor, i.e., the cross-spectrum is entirely determined by the timescale of the correlated AMPA receptors. (**E**) Same as panel C, but for the case where only AMPA receptors have been correlated. Notice that now the correlations boost the amplitude of the AMPA receptor timescale in the ensemble signal. (**F**) When all synapses are correlated, the CSD is equal to the sum of the GABAR only CSD (panel B) and the AMPAR only CSD (panel D) (**G**) Correlating all synapses boosts the amplitude of both the AMPA and GABA receptor timescales. (**H**) Based on panels B-G, the CSD between two neurons was modelled as a weighted sum of their GABAR CSD (panel B) and their AMPAR CSD (panel D). The CSD amplitudes decreased with distance following a Gaussian function with variance $\sigma^{2}$ such that nearby neurons were more correlated than distant neurons. Illustration adapted from Brake et al. [3]. (**I**). The ensemble EEG spectrum was modelled while separately varying the spatial decay for the GABAR component ($\sigma_{GABAR}^{2}$) and AMPAR component ($\sigma_{AMPAR}^{2}$) of the CSD. The data in Fig 5B was captured with $\sigma_{GABAR}^{2}=0.1$ mm^2^ and $\sigma_{AMPAR}^{2}=0.005$ mm^2^.(TIF)

S8 FigCorrelation timescale influences the spectral peak width of apEEG rhythm.(**A**) Plot of Eq 6 for $\sigma_{t}=60$ *ms*. (**B**) Same as in Fig 6G, but for $\sigma_{t}=60$ *ms*. (**C**) Same as in Fig 6B, but for $\sigma_{t}=60$ *ms*. (**D–F**) Same as in A-C, but for $\sigma_{t}=4$ *ms*.(TIF)

S9 FigAPs synchronized over shorter distances still explain high frequency oscillations.Same as Fig 7C, except with apEEG simulated with $\sigma_{x}=1$ *mm* instead of 3*mm*.(TIF)

S10 FigSchematic of dendrite asymmetry index calculation.(**A**) Example morphology of a layer 6 pyramidal cell. Note that the diameter of the dendrites have been increased by a factor of two in the figure to better illustrate the variation in dendrite diameter throughout the arbour. (**B**) Zoomed in view of the indicated dendritic branch, showing that the dendrite morphology is represented by truncated cone segments.The diameter and length of each truncated cone is printed. For illustrative purposes, the dendrite diameter is drawn with a scaling factor of 10. The same dendrite segment with correct proportions is shown in the insert for comparison. (**C**) To calculate the dendrite asymmetry index (Eq 17), each segment is represented by its midpoint in space (${\text{x}}_{i}$) and its total volume ($V_{i}$), calculated as $1∕3\pi L(r_{1}^{2}+r_{1}r_{2}+r_{2}^{2})$. The black dots plotted at the midpoint of each dendrite segment are scaled proportionally to the segment’s volume. (**D**) The black dots represent the midpoints and their sizes represent the volume of all dendrite segments. The red star indicates the result of the asymmetry index calculation (Eq 17), prior to taking the Euclidean norm. The length of the red line is thus the asymmetry index of this neuron. For illustrative purposes, the equation result has been scaled here by 0.01 as otherwise the vector would be too long to depict. Note, however, that the regression in Fig 3 holds for any arbitrary scaling of Eq 17.(TIF)

S1 Table List of 68 neuron models used in Fig Fig 2. Nomenclature from Markram et al. [1].
(XLXS)

S2 Table List of morphology classes used in Fig 3. Nomenclature from Markram et al. [1].
(XLXS)
