# Supplementary figures and images for: Contributions of action potentials to scalp EEG: Theory and biophysical simulations
Source: PLoS Comput Biol. 2025 Feb 4;21(2):e1012794. doi: 10.1371/journal.pcbi.1012794 (PMC11809874; doi:10.1371/journal.pcbi.1012794)

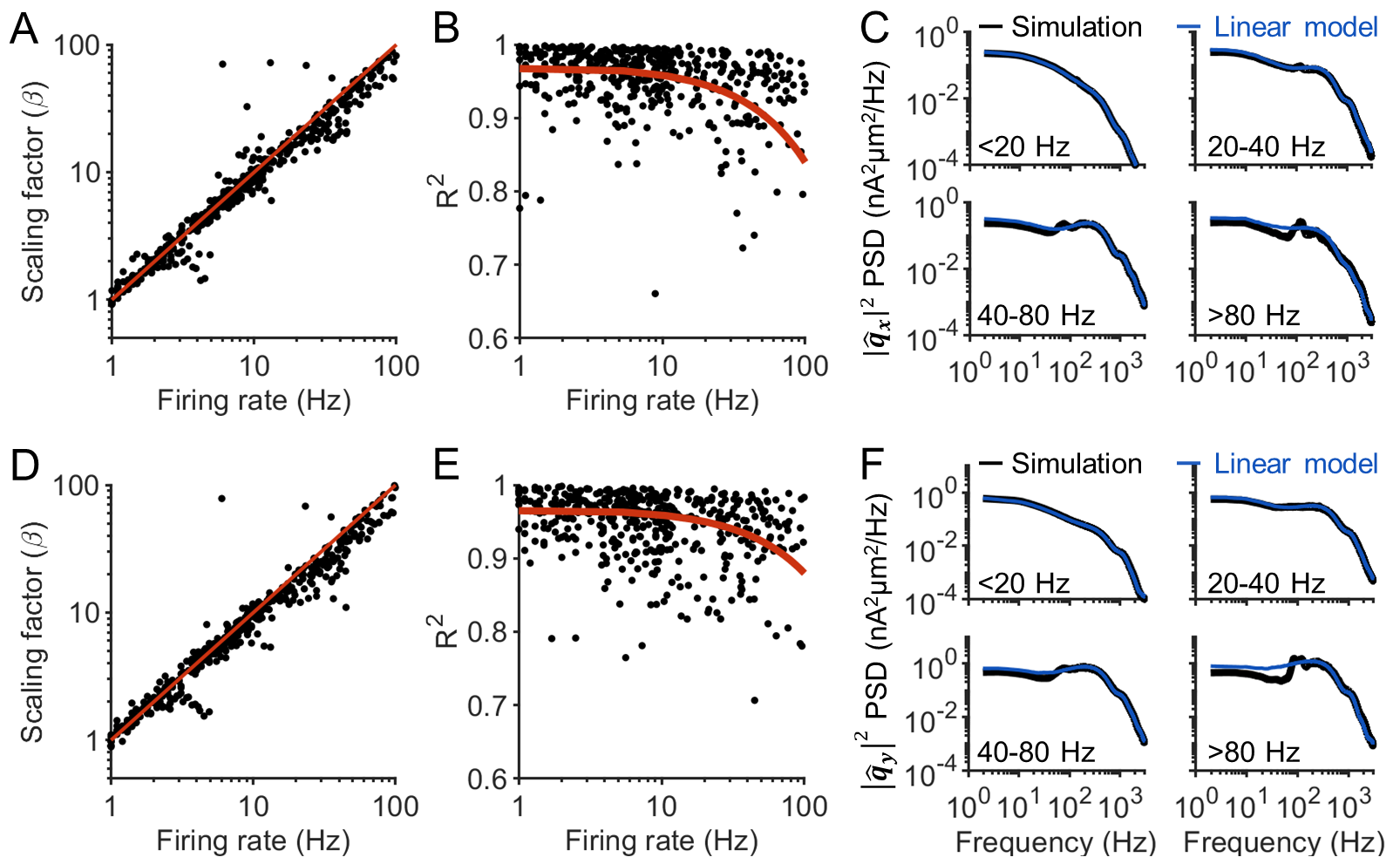

Supplement: S1 Fig — (A–C) Same as Fig 2, but for the x component of the single-neuron dipole. (D-F) Same as Fig 2, but for the y component of the single-neuron dipole. (TIF) [file pcbi.1012794.s001.tif]

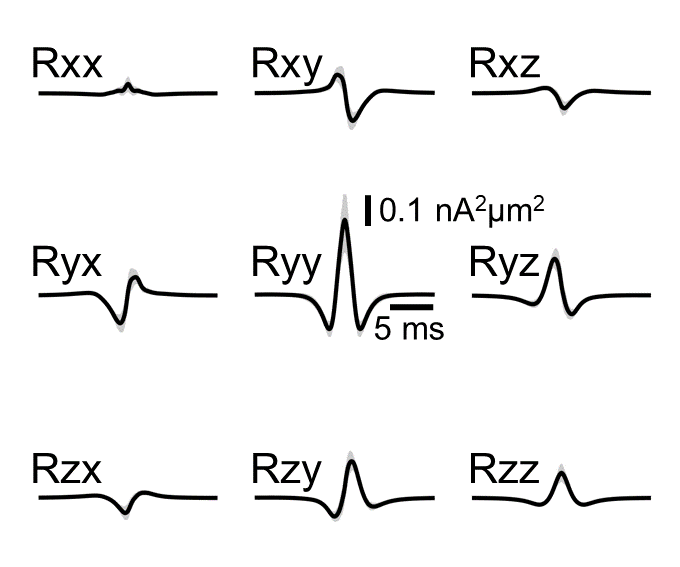

Supplement: S2 Fig — Solid black line indicates average across all pairs of 1035 neuron models, weighted by the relative abundance of the pairing (Fig 3B). Shading reflects 95% confidence interval of the mean. (TIF) [file pcbi.1012794.s002.tif]

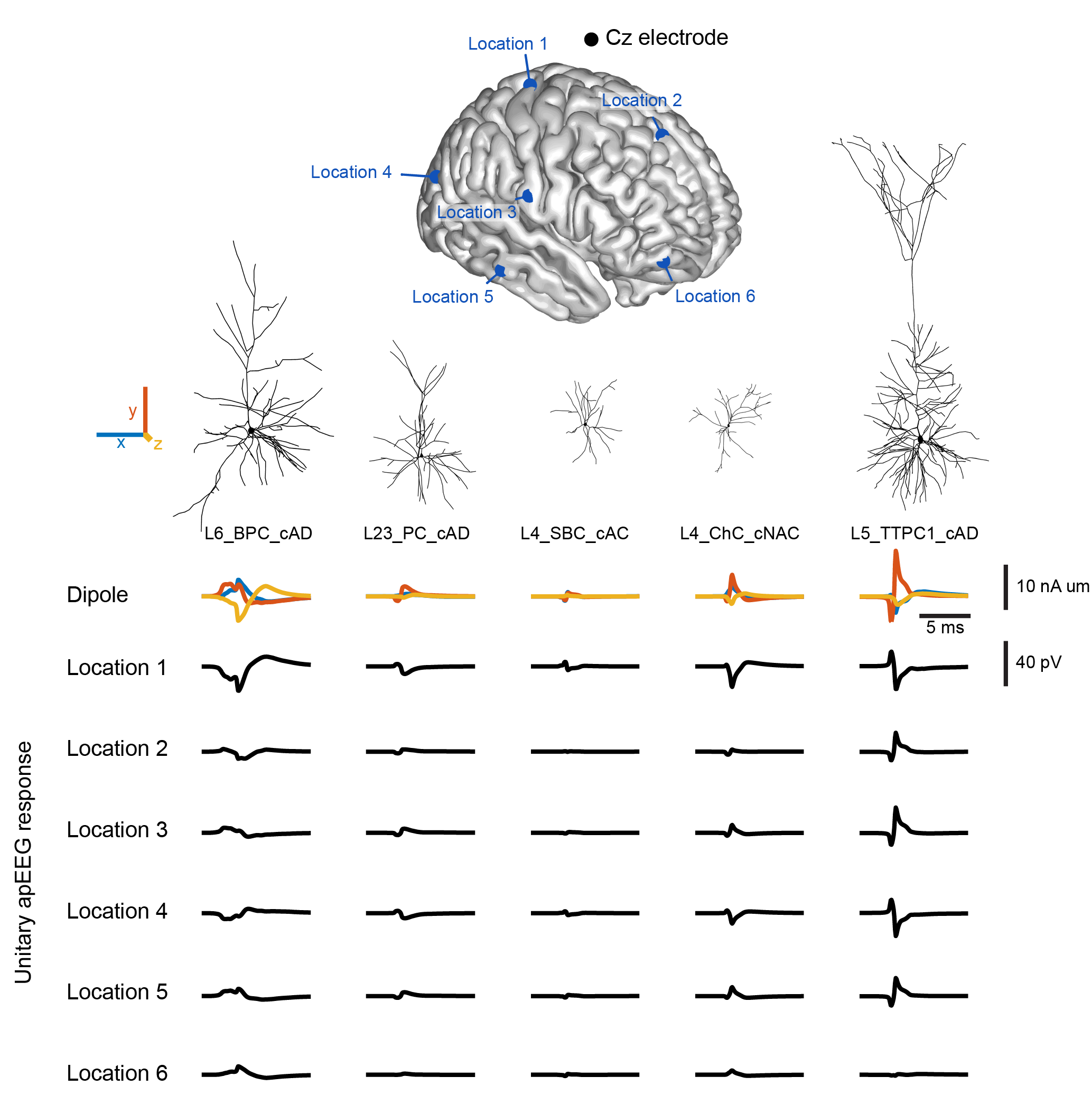

Supplement: S3 Fig — The source locations are labeled on the brain template at the top of the figure. The lines extending from each labeled location indicate the direction of the respective normal vector, which were used to orient the apical-basal axes of the neurons. The morpohlogies of the five neuron models are shown below the cortical template and are labeled using the syntax from Markram et al. [34]. From left to right, these models represent a layer 6 pyramidal neuron, a layer 2/3 pyramidal neuron, a layer 4 basket cell, a layer 4 chandelier cell, and a layer 5 thick-tufted pyramidal neuron. Below each neuron morphology is shown the x (blue), y (red), and z (orange) components of the single-neuron dipole in response to an AP. Finally, at the bottom of the figure is shown the unitary apEEG responses produced at the Cz electrode when each neuron model is placed at each of the six indicated cortical locations. Note that amplitude of the apEEG signal depends on the overall size of the neuron and the distance from the neuron to the electrode. (TIF) [file pcbi.1012794.s003.tif]

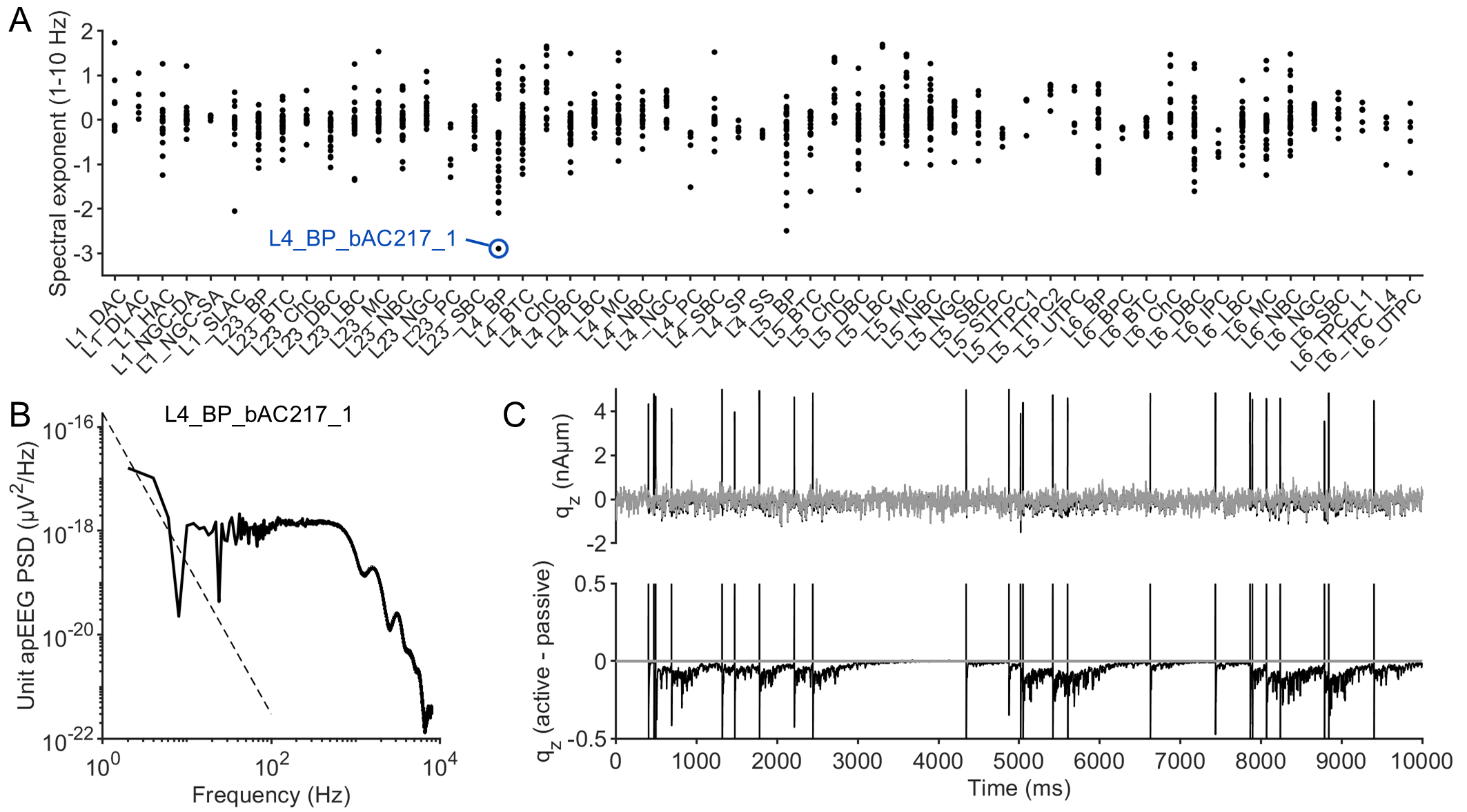

Supplement: S4 Fig — (A) The slope of the unitary AP spectrum for every neuron model, calculated between 1-10 Hz. A neuron (ID: L4_BP_bAC217_1) with a particularly negative slope is indicated in blue. (B) Power spectrum of the unitary AP spectrum for the neuron indicated in panel A, with 1/f trend fitted at low frequencies (dashed black line). (C) Top: z component of the single-neuron dipole of the neuron indicated in panel A (black) and with somatic and axonal sodium channels removed to generate a passive model (grey). Bottom: Difference between the active and passive neuron models. Note that the spikes have been truncated at ± 0 . 5 nA�m. After each spike, the active model’s dipole takes hundreds of milliseconds to reconverge to the passive model. The same phenomena were observed in the dipoles x and y components. (TIF) [file pcbi.1012794.s004.tif]

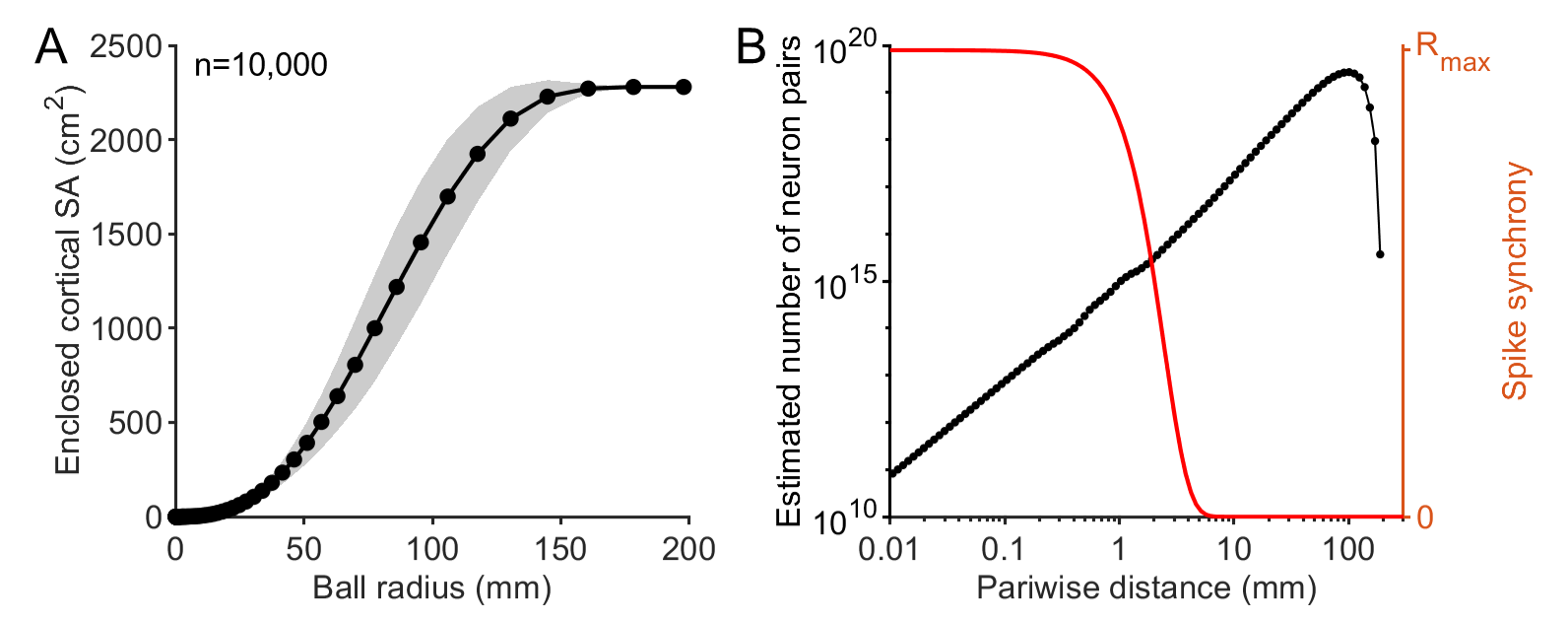

Supplement: S5 Fig — (A) Surface area (SA) of the cortex from New York head model enclosed within balls of increasing radii, with the origin of the ball placed at 10,000 cortical locations. Black dots indicate the discrete ball radii for which the surface area was calculated. Shading reflects standard deviation across the 10,000 starting points. (B) The derivative of the surface area with respect to radius (black), scaled to obtain the density of neuron pairs for each pairwise distance (see Methods). The red curve illustrates the coupling kernel, as in Fig 4A. The vast majority of neuron pairs are separated by more than 10 mm and are therefore not correlated in the model. (TIF) [file pcbi.1012794.s005.tif]

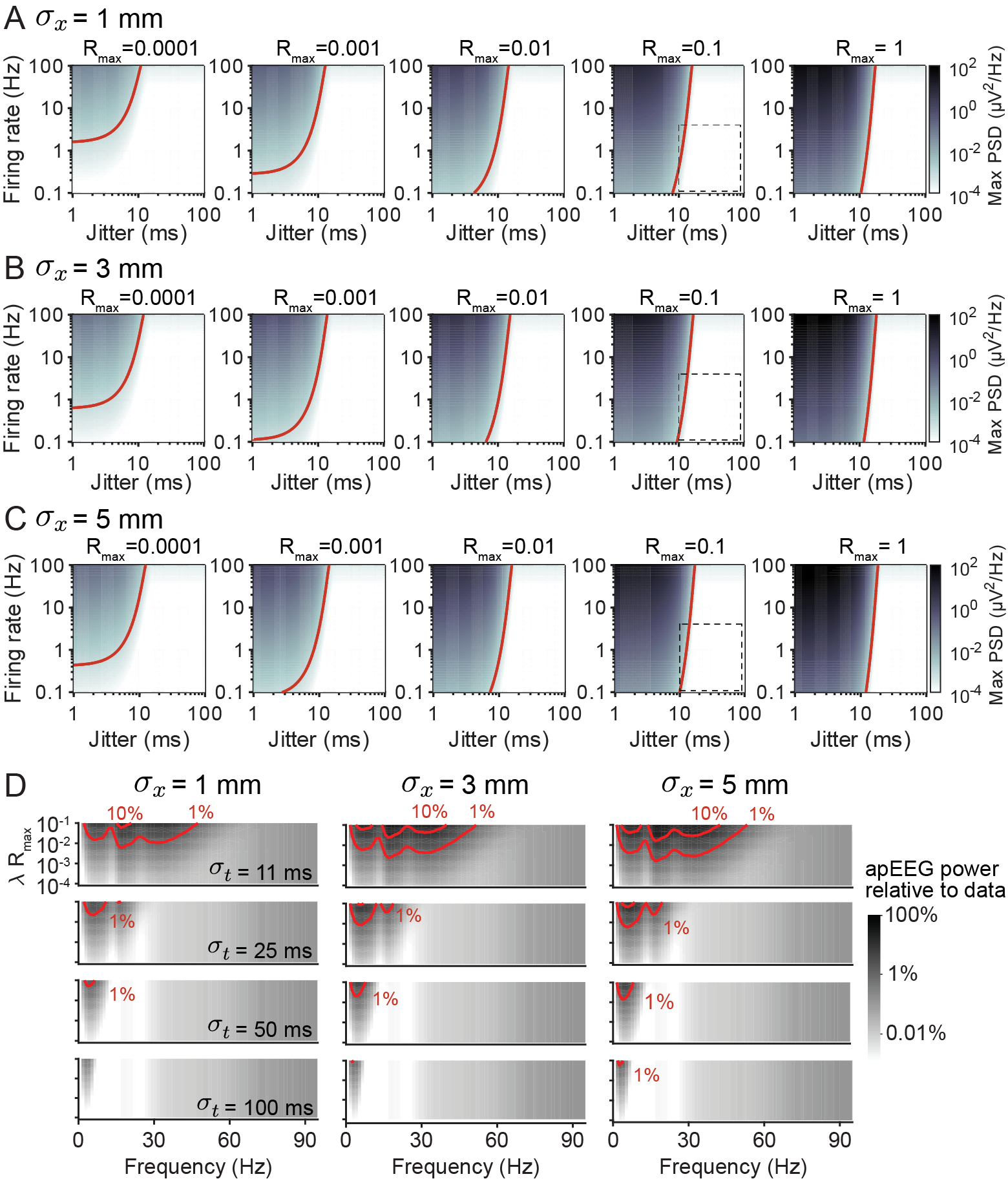

Supplement: S6 Fig — (A) Same as in Fig 4F, but for σx2=1 mm2. (B) Same as in Fig 4F. (C) Same as in Fig 4F, but for σx2=5 mm2. (D) Left: Same as in Fig 5D, but for σx2=1 mm2. Middle: Same as in Fig 5. Right: Same as in Fig 5D, but for σx2=5 mm2 (left). (TIF) [file pcbi.1012794.s006.tif]

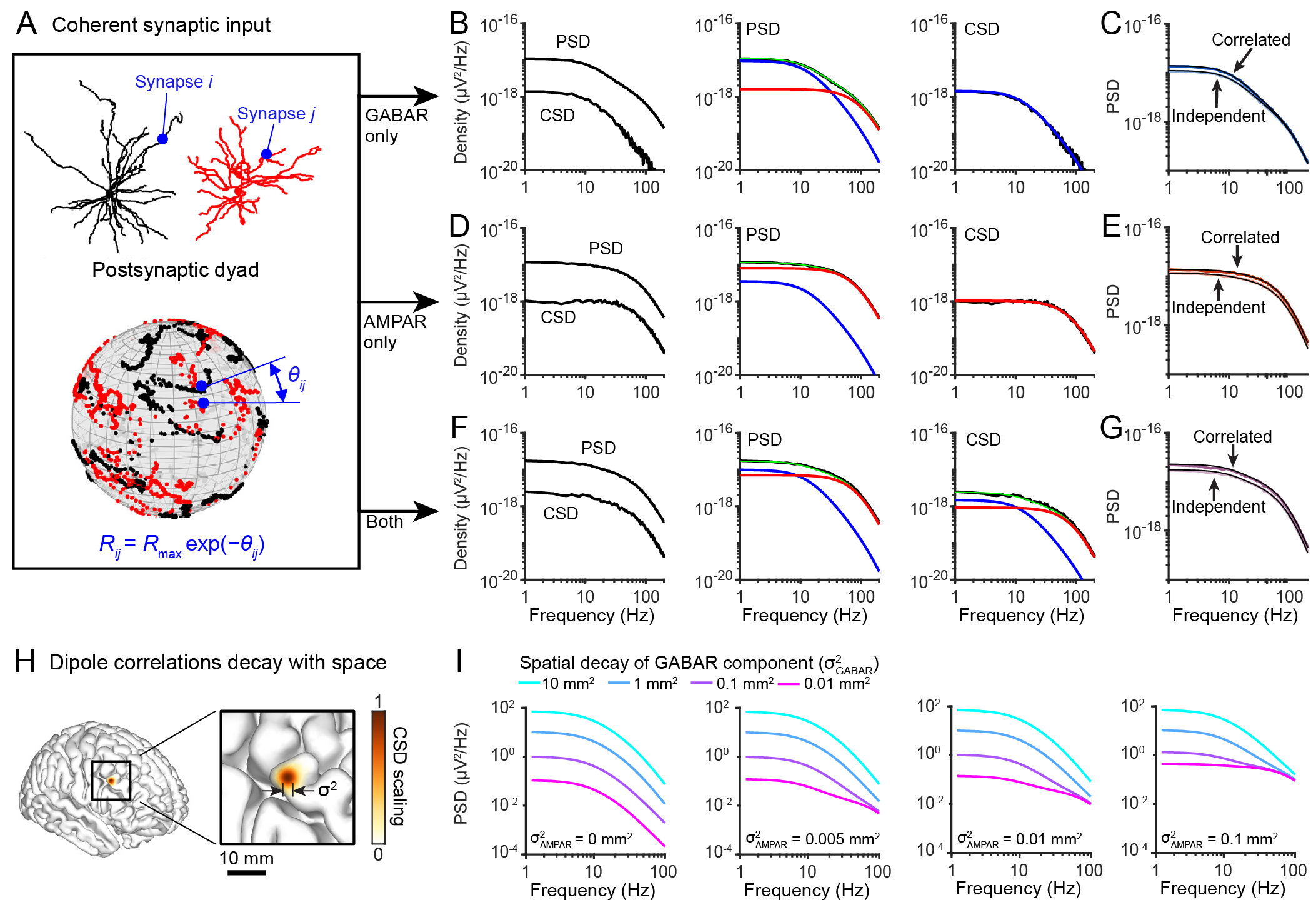

Supplement: S7 Fig — (A) Illustration from Brake et al. [3] of the minimal model for dipole coherence between two neurons. Briefly, synapses on the two neurons are activated with correlated Poisson processes, with correlation strengths determined by the angular distance between the synapses. See Brake et al. [3] for more details. (B) Only GABA receptor activation has been correlated. Left: the average power spectral density (PSD) and cross spectral density (CSD) for the two single-neuron EEGs. Middle: The PSD has been fit with the sum of two Lorentzian functions (Eq 18) (green). The GABA receptor (blue) and AMPA receptor (red) related timescales are also shown. Right: The CSD is entirely fit with the GABA receptor timescale from the middle panel. In other words, the cross spectrum is entirely determined by the timescale of the correlated GABA receptors. (C) The PSD of the summed single-neuron EEGs from the two neurons when the synapses were correlated and independent. Both PSDs were fit with Eq 18 (black lines) using the same τI1, τI2, τE1, and τE2 as the PSD in panel B. As expected from the CSD, the difference between the correlated and independent ensemble EEG was captured by increasing the scaling of the GABA receptor timescales. (D) Same as panel B, but here only AMPA receptor activation has been correlated. Now the CSD (right) is captured entirely by the timescale of the AMPA receptor, i.e., the cross-spectrum is entirely determined by the timescale of the correlated AMPA receptors. (E) Same as panel C, but for the case where only AMPA receptors have been correlated. Notice that now the correlations boost the amplitude of the AMPA receptor timescale in the ensemble signal. (F) When all synapses are correlated, the CSD is equal to the sum of the GABAR only CSD (panel B) and the AMPAR only CSD (panel D) (G) Correlating all synapses boosts the amplitude of both the AMPA and GABA receptor timescales. (H) Based on panels B-G, the CSD between two neurons was modelled as a weighted sum of [file pcbi.1012794.s007.tif]

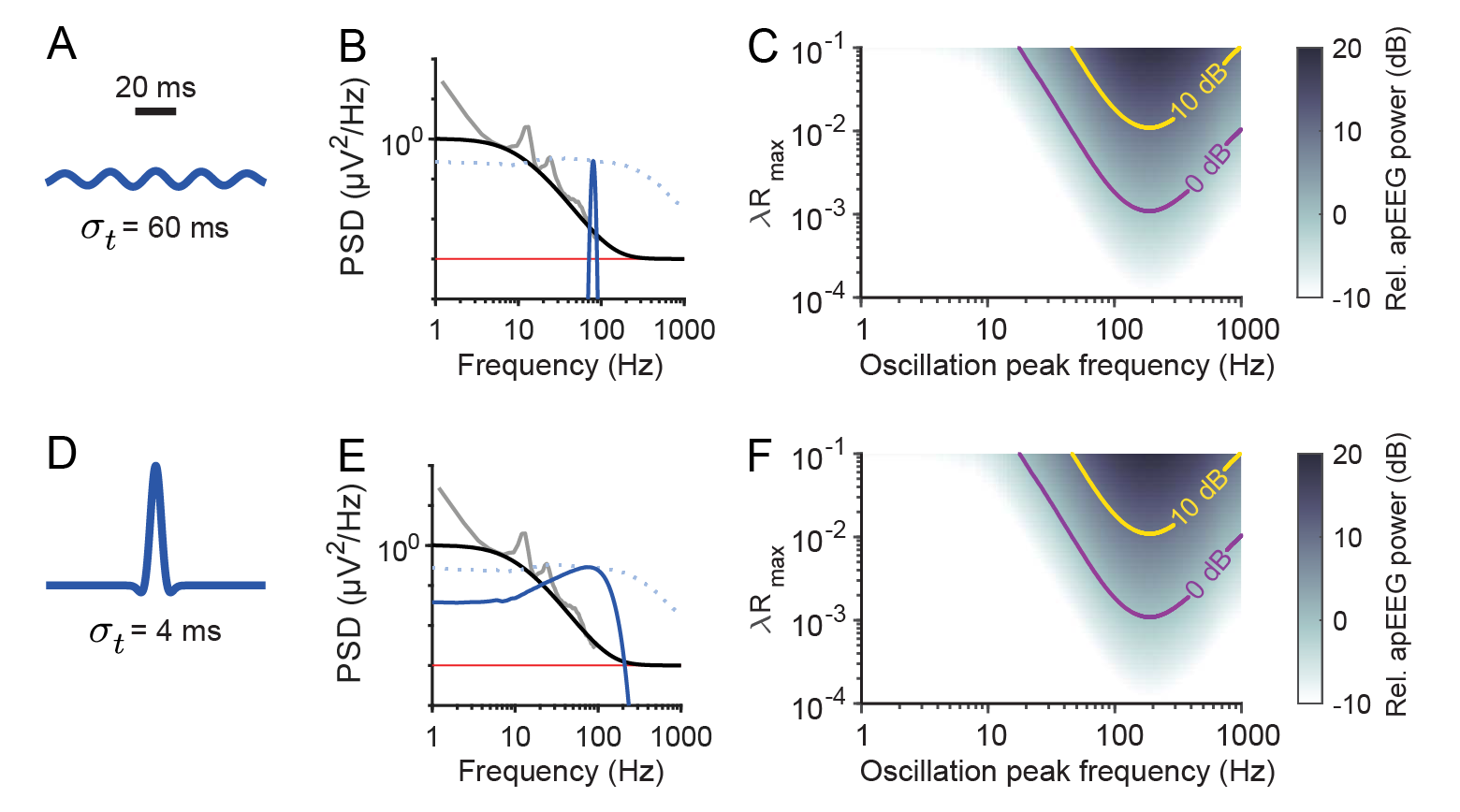

Supplement: S8 Fig — (A) Plot of Eq 6 for σt=60 ms. (B) Same as in Fig 6G, but for σt=60 ms. (C) Same as in Fig 6B, but for σt=60 ms. (D–F) Same as in A-C, but for σt=4 ms. (TIF) [file pcbi.1012794.s008.tif]

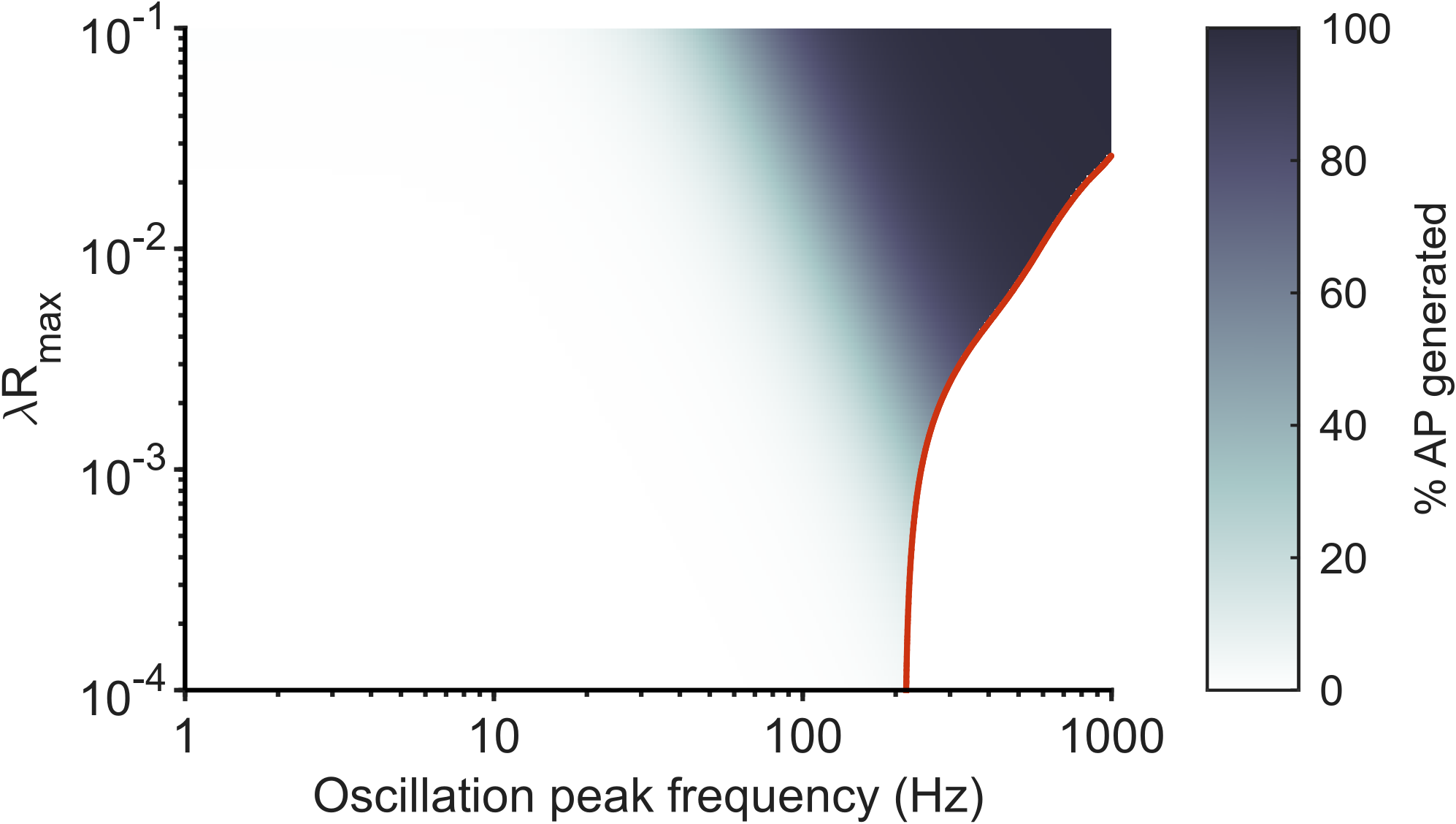

Supplement: S9 Fig — Same as Fig 7C, except with apEEG simulated with σx=1 mm instead of 3mm. (TIF) [file pcbi.1012794.s009.tif]

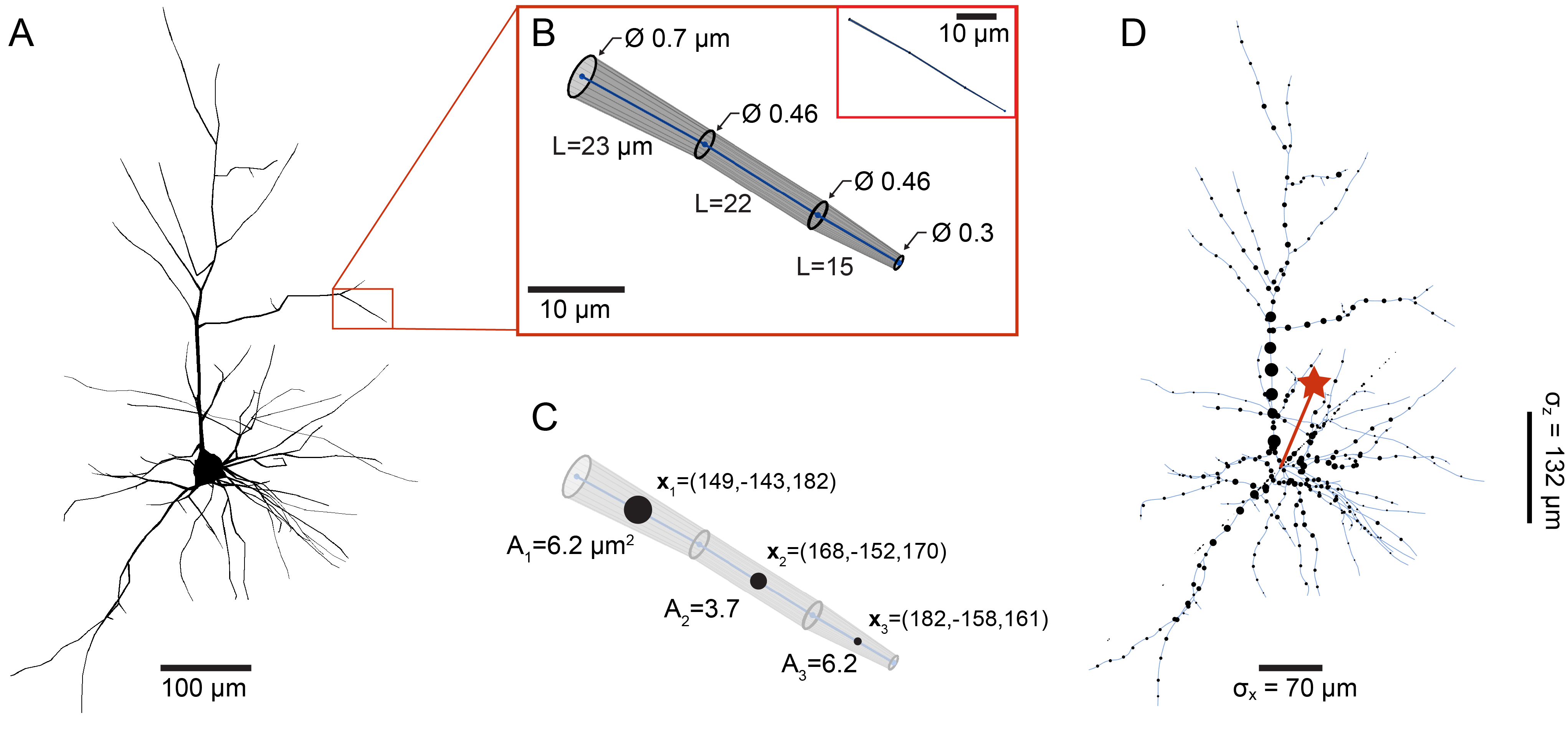

Supplement: S10 Fig — (A) Example morphology of a layer 6 pyramidal cell. Note that the diameter of the dendrites have been increased by a factor of two in the figure to better illustrate the variation in dendrite diameter throughout the arbour. (B) Zoomed in view of the indicated dendritic branch, showing that the dendrite morphology is represented by truncated cone segments.The diameter and length of each truncated cone is printed. For illustrative purposes, the dendrite diameter is drawn with a scaling factor of 10. The same dendrite segment with correct proportions is shown in the insert for comparison. (C) To calculate the dendrite asymmetry index (Eq 17), each segment is represented by its midpoint in space (xi) and its total volume (Vi), calculated as 1∕3πL(r12+r1r2+r22). The black dots plotted at the midpoint of each dendrite segment are scaled proportionally to the segment’s volume. (D) The black dots represent the midpoints and their sizes represent the volume of all dendrite segments. The red star indicates the result of the asymmetry index calculation (Eq 17), prior to taking the Euclidean norm. The length of the red line is thus the asymmetry index of this neuron. For illustrative purposes, the equation result has been scaled here by 0.01 as otherwise the vector would be too long to depict. Note, however, that the regression in Fig 3 holds for any arbitrary scaling of Eq 17. (TIF) [file pcbi.1012794.s010.tif]
